# Cellular cartography: Towards an atlas of the neuronal microtubule cytoskeleton

**DOI:** 10.3389/fcell.2023.1052245

**Published:** 2023-03-22

**Authors:** Malina K. Iwanski, Lukas C. Kapitein

**Affiliations:** Cell Biology, Neurobiology and Biophysics, Department of Biology, Faculty of Science, Utrecht University, Utrecht, Netherlands

**Keywords:** microtubules, neurons, microtubule stability, post-translational modifications, microtubule-associated proteins, microscopy

## Abstract

Microtubules, one of the major components of the cytoskeleton, play a crucial role during many aspects of neuronal development and function, such as neuronal polarization and axon outgrowth. Consequently, the microtubule cytoskeleton has been implicated in many neurodevelopmental and neurodegenerative disorders. The polar nature of microtubules is quintessential for their function, allowing them to serve as tracks for long-distance, directed intracellular transport by kinesin and dynein motors. Most of these motors move exclusively towards either the plus- or minus-end of a microtubule and some have been shown to have a preference for either dynamic or stable microtubules, those bearing a particular post-translational modification or those decorated by a specific microtubule-associated protein. Thus, it becomes important to consider the interplay of these features and their combinatorial effects on transport, as well as how different types of microtubules are organized in the cell. Here, we discuss microtubule subsets in terms of tubulin isotypes, tubulin post-translational modifications, microtubule-associated proteins, microtubule stability or dynamicity, and microtubule orientation. We highlight techniques used to study these features of the microtubule cytoskeleton and, using the information from these studies, try to define the composition, role, and organization of some of these subsets in neurons.

## Introduction

From the mistaken identification of microtubules in electron micrographs as “long, tubular elements of the endoplasmic reticulum” in the dendrites of neurons (Palay, 1956), to the high-resolution studies of microtubules presently being conducted, the scientific exploration of microtubules has long been a visual one; advances in techniques have furthered our understanding of microtubule diversity, organization, and function, and reciprocally, our desire to further this understanding has driven technological advancements.

Shortly after the naming of microtubules (Ledbetter and Porter, 1963; Slautterback, 1963), studies of colchicine prompted the identification of tubulin as the subunit comprising these polymeric fibrils (Borisy and Taylor, 1967a; Borisy and Taylor, 1967b). Advances in electron microscopy (EM) and fixation methods subsequently allowed us to postulate how tubulin subunits are arranged within a microtubule (Tilney et al., 1973). Since then, countless studies have given us a detailed picture of the different conformations of tubulin, the structure of growing and shrinking microtubules, and the architecture of the microtubule network in cells.

The quasi-helical symmetry of microtubules allowed for the averaging of tubulin structures within the lattice, facilitating early high resolution EM studies. Advances in EM imaging and analysis, as well as clever tricks such as the decoration of microtubules with kinesin to break the symmetry within the lattice and the use of nucleotide analogs, have allowed scientists to determine the structure of different conformations of tubulin with impressive resolution (see, e.g., (Alushin et al., 2014; Manka and Moores, 2018; LaFrance et al., 2022). Thus, we now know that microtubules are approximately 25 nm wide hollow tubes comprised, in our cells, of 13 laterally-associated protofilaments, each of which is a linear chain of α,β-tubulin heterodimers polymerized head-to-tail to produce filaments with two distinct ends. Both tubulin monomers are structurally similar with a globular body of ∼4 nm diameter that binds GTP and makes contacts with neighbouring tubulin dimers in the microtubule lattice, and a flexible C-terminal tail located at the outside surface of the microtubule. However, only β-tubulin, exposed at the more dynamic plus-ends of microtubules (Mitchison, 1993), hydrolyzes and exchanges its GTP in a hydrolysis cycle intricately coupled to conformational changes in the tubulin dimer (Alushin et al., 2014; Manka and Moores, 2018; LaFrance et al., 2022). These conformational changes facilitate the growth and shrinkage of microtubules; GTP-tubulin incorporates into the microtubule at the growing end and undergoes GTP hydrolysis within the microtubule lattice after some delay, resulting in the presence of a stabilizing cap of GTP-tubulin. If this cap is lost and GDP-tubulin is exposed instead, the microtubule undergoes a catastrophe and rapidly depolymerizes unless it is rescued and re-enters a period of growth. These cycles of polymerization and depolymerization are known as microtubule dynamic instability (Mitchison and Kirschner, 1984).

Preceding structural studies, observations of microtubule dynamic instability are precisely what initially allowed scientists to posit that microtubules are polar; the plus- and minus-ends of microtubules were defined based on their growth speeds; the fast-growing end was termed the plus-end, while the slow-growing end was called the minus-end (Borisy, 1978). While these older studies focused on the distinct biochemical properties of the plus- and minus-end (Allen and Borisy, 1974; Dentler et al., 1974; Bergen and Borisy, 1980; Mitchison, 1993), this polarity arises—importantly—from the head-to-tail association of α,β-tubulin heterodimers within the microtubule and can thus be read along the lengths of these filaments. This allows them to serve as tracks for transport: motor proteins of the kinesin family and dynein can use the asymmetry of these filaments to walk in a directed manner towards either the plus- or minus-end of the filament. Thus, it has long been assumed that the polarity of microtubules is carefully controlled in cells as this would determine the direction of transport. Indeed, we now know that the dynamics and positioning of microtubules can be regulated by a slew of nucleation factors and other microtubule-associated proteins (MAPs) that influence the nucleation, growth, shrinkage, stability, and organization of microtubules. By specifically utilizing different MAPs, cells are able to construct unique architectures of microtubules that are essential for their function. Cycling cells typically have a radial array of microtubules focused at a centrosomal microtubule organizing center (MTOC) or two overlapping asters of microtubules during cell division. Post-mitotic cells such as neurons, on the other hand, tend to have polar arrays of microtubules, likely important to establish and maintain cell polarity and function, at least in part by dictating the transport of cargoes in the cell by kinesins and dynein (Kapitein and Hoogenraad, 2015; Burute and Kapitein, 2019).

Some MAPs, instead of (strictly) controlling microtubule organization or dynamics, regulate kinesin- or dynein-driven transport by inhibiting or activating some of these motor proteins; this forms the basis of the so-called MAP code (Monroy et al., 2020). Importantly, not all motors are similarly affected by these MAPs. For example, while tau and MAP2 have inhibitory effects on both kinesin-1 and kinesin-3 in *in vitro* reconstitution experiments, MAP7 recruits and activates only kinesin-1, and MAP9 and Septin 9 (SEPT9) activate only kinesin-3 (Karasmanis et al., 2018; Hooikaas et al., 2019; Monroy et al., 2020). Furthermore, doublecortin (DCX) and doublecortin-like kinase 1 (DCLK1) also have inhibitory effects on kinesin-1, but both permit kinesin-3 motility (Monroy et al., 2020). Interestingly, while most MAPs seem to have a limited effect on dynein, MAP9 does inhibit its motility (Monroy et al., 2020). In this way, MAPs localized to different places in the cell can help guide cargoes bound to different motor proteins to their correct destination.

Another aspect potentially controlling microtubule-based transport by kinesins and dynein is the so-called tubulin code or the diversity of tubulin. Our cells encode nine genes for α-tubulin and nine genes for β-tubulin (Janke and Magiera, 2020). These isotypes differ largely in terms of their C-terminal tails, but there is also variability in the M-loop (Roll-Mecak, 2020), a structural component essential for lateral interactions between neighbouring tubulin dimers (Alushin et al., 2014). Of these isotypes, at least five of each are expressed at some point during neuronal development (Hausrat et al., 2020). Moreover, tubulin is subject to a wide array of post-translational modifications (PTMs) that further increase the diversity of the tubulin pool in our cells. Many of these PTMs occur on the C-terminal tails of the tubulin dimer such as the removal of the terminal tyrosine (detyrosination) (Arce et al., 1975; Hallak et al., 1977; Gundersen et al., 1984) or additionally of the adjacent glutamate (∆2) (Paturle-Lafanechere et al., 1991), but some also occur on the body of tubulin. The best-studied example of this is the acetylation of Lysine 40 on α-tubulin (αK40). This residue resides inside the microtubule lumen and its acetylation is thought to be a marker for long-lived, stable microtubules (Schulze et al., 1987). This PTM is well-studied in part because of kinesin-1’s strong preference for acetylated microtubules in cells (Reed et al., 2006; Cai et al., 2009; Tas et al., 2017); however, the motor is indifferent to this modification *in vitro* (Walter et al., 2012; Kaul et al., 2014). This suggests that kinesin-1 may not be recognizing a specific feature of acetylated microtubules directly, but rather that the microtubules that kinesin-1 prefers are typically also acetylated in cells. Alternatively, some (structural) aspect of acetylated microtubules in cells may be lost *in vitro*, for example, due to the use of Taxol, a drug that binds to microtubules and suppresses their dynamics, or GMPCPP, a non-hydrolyzable GTP analog, to stabilize microtubules. Together, the tubulin isotypes and their PTMs make up the so-called tubulin code (Janke and Magiera, 2020; Park and Roll-Mecak, 2018; Roll-Mecak, 2020).

Microtubules composed of different tubulin isotypes, bearing a variety of PTMs, and decorated by a slew of MAPs are organized into a spectacularly asymmetric array of microtubules in neurons. The organization of the neuronal microtubule cytoskeleton is thought to be essential for neuronal polarity, a prerequisite for their function. Thus, much scientific effort has focused on understanding precisely how the microtubule cytoskeleton in neurons is organized and in dissecting the processes that allow this network to be built and maintained. In this review, we will highlight important studies on the architecture of the neuronal microtubule cytoskeleton and the technological advancements that have facilitated these discoveries. Specifically, we will focus on the heterogeneity of microtubules and aim to define neuronal microtubule subsets, including their characteristics and functional roles. We will also speculate on how different aspects defining subsets can be coordinated and how distinct subsets can be maintained simultaneously in the cell.

## Determining microtubule orientation

In neurons, the differential localization of cargoes to the axon and dendrites is essential for neuronal polarity and likely relies on the directed transport of these cargoes by microtubule-based motor proteins that can enter the axon and/or the dendrites (Lipka et al., 2016). How this transport is directed is a multi-faceted problem, but fundamentally depends on the orientation of microtubules in the neurites: if microtubules are oriented plus-end-out, plus-end-directed motor proteins such as kinesin-1 and kinesin-3 will drive anterograde (away from the cell body) transport, while minus-end-directed motor proteins such as dynein will drive retrograde (towards the cell body) transport; however, if the orientation of the microtubules is reversed, the opposite holds true. Thus, it is important to consider how microtubules are oriented in neurons.

Calculations based on the diffraction patterns from electron micrographs of microtubule doublets provided an early indication that microtubules are polar structures—albeit with some uncertainty (Amos and Klug, 1974). Since then, a variety of techniques have facilitated the visualization of microtubule orientation both *in vitro* and in cells.

### Molecular arrowheads

One technique to determine the orientation of a polar filament is to decorate it with a molecular arrowhead. That is, to decorate the filament with some other component that is asymmetrical on a larger scale that is resolvable with a given imaging method. In the actin field, myosin heads decorating actin filaments fulfilled precisely this role; in electron micrographs, myosin appeared as arrowheads (or chevrons) bound along actin filaments with a uniform orientation, suggesting that the underlying filaments were also polar along their length (Huxley, 1963). The appearance of these decorated actin filaments was also responsible for the nomenclature of the two ends of the filament: “pointed” and “barbed.”

**FIGURE 1:**
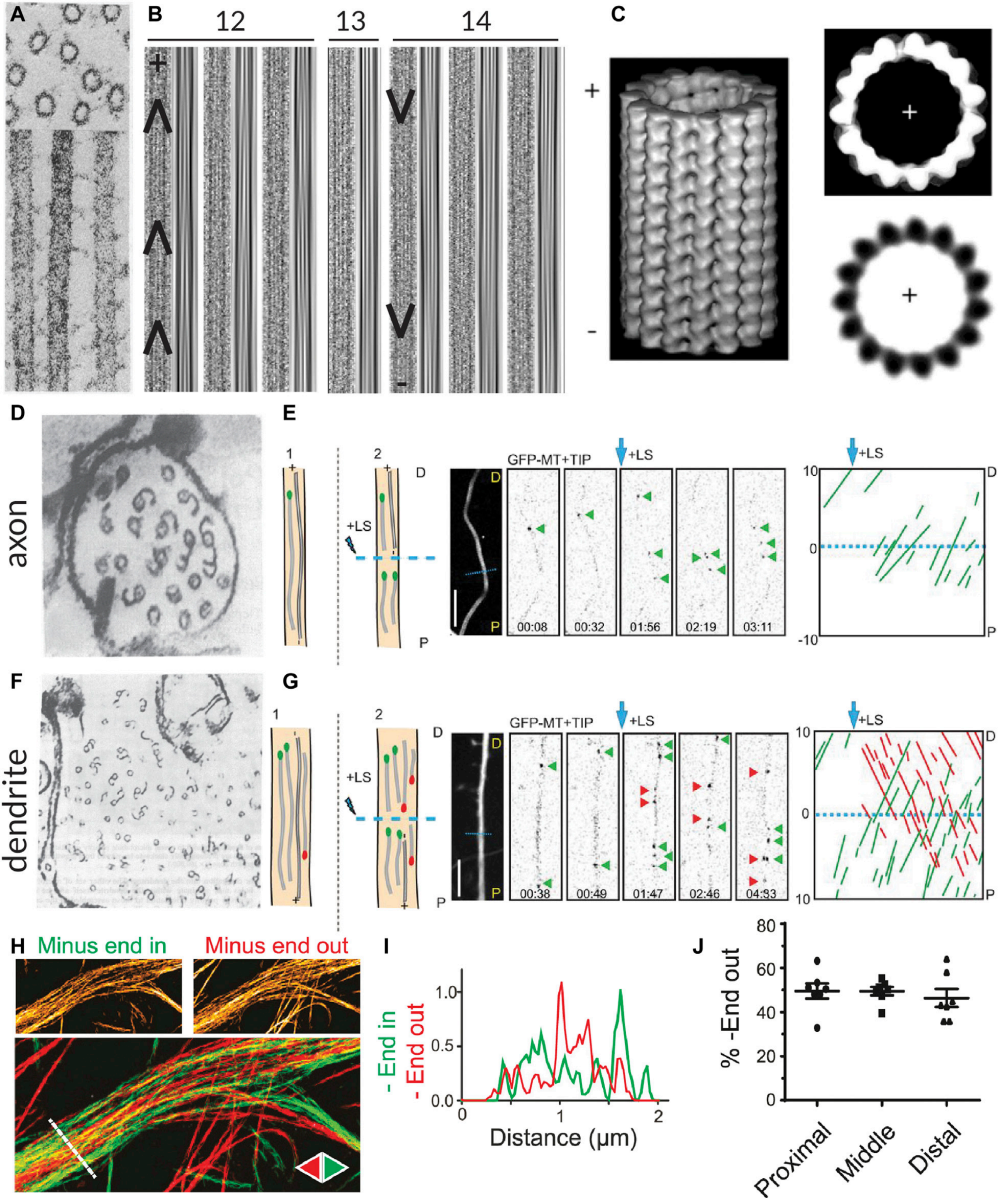
Methods for visualizing the polarity of (neuronal) microtubules. **(A)** Decoration of microtubules polymerized from porcine brain tissue with dynein heavy chain fragments. Note the uniform tilt of the dynein fragments along the length of a given microtubule, indicating the structural polarity of the filaments. Taken from (Murphy and Borisy, 1975). **(B)** The moiré patterns from microtubules grown from purified calf brain tubulin and nucleated off of isolated centrosomes such that their polarity is known. All microtubules oriented plus-end-up. Chevrons drawn in to help visualize the chevrons in the moiré patterns. When microtubules have 13 protofilaments they run parallel to the microtubule axis, but when they have another number of protofilaments, they have a supertwist, producing arrowheads of a uniform direction; however, the direction of these arrowheads depends on the number of protofilaments, which dictates the handedness of the supertwist. Taken from (Chrétien et al., 1996). **(C)** A 3D reconstruction of a 15 protofilament microtubule, as well as a corresponding axial view and a projection of a thin slice from this reconstruction, both viewed from the plus-end towards the minus-end. Note that the tubulin monomers are tilted counter-clockwise when viewed from this end. This tilt is independent of protofilament number. Taken from (Sosa and Chrétien, 1998). **(D)** Cross-section of an axon from a rat hippocampal neuron with microtubules subject to hook decoration. Neurons are lysed and a hooking buffer containing additional brain tubulin is added such that it forms hooks along the existing microtubules. Note that in this axon viewed from the growth cone towards the cell body, all the hooks are oriented clockwise, indicating that the plus-ends of the microtubules are pointing towards the growth cone; however, many microtubules do not have any hooks, so their orientation is unknown. Taken from (Baas et al., 1988). **(E)** Diagram, confocal snapshots, and kymograph from a laser severing and EB comet tracking experiment in an axon (proximal end pointing downwards) from a rat hippocampal neuron. In the diagram, stable microtubules have no EB comet preceding laser severing, while following laser severing, a microtubule with an EB comet can grow from the stable template. Position and time of laser severing indicated in blue. The kymograph shows that all comets move anterogradely both before and after laser severing, but the frequency of comets increases substantially after severing. Taken from (Yau et al., 2016). **(F)** Cross-section of part of a dendrite from a rat hippocampal neuron with microtubules subject to hook decoration as in **(D)**. Note that in this dendrite viewed from the growth cone, some hooks are pointing clockwise, while others are pointing counter-clockwise, indicating that microtubules are of mixed orientation. Again, many microtubules do not have hooks, so their orientation is unknown. Taken from (Baas et al., 1988). **(G)** Diagram, confocal snapshots, and kymograph from a laser severing and EB comet tracking experiment in a dendrite (proximal end pointing downwards) from a rat hippocampal neuron. In the dendrite, there are EB comets moving both anterograde (green) and retrograde (red), as seen in the snapshots and the kymographs. Again, the comet frequency increases massively after laser severing. Taken from (Yau et al., 2016). **(H)** motor-PAINT reconstruction from a dendrite in a rat hippocampal neuron. Microtubules reconstructed in green had kinesin motors moving from left (proximal) to right (distal) and are thus plus-end-out, while those coloured red are pointing in the opposite direction. Taken from (Tas et al., 2017). **(I)** The intensity profiles along the line indicated in **(H)**, showing that minus-end-out microtubules are bundled centrally, while plus-end-out (minus-end-in) microtubules are bundled peripherally. Taken from (Tas et al., 2017). **(J)** A graph showing the quantification of the percentage of microtubules (based on motor localizations) that are oriented minus-end-out in the proximal, middle, and distal parts of dendrites. This percentage is always close to 50%, suggesting that microtubule orientations are mixed 50–50 along the length of the dendrites. Taken from (Tas et al., 2017).

What we now know to be axonemal dynein fragments are similarly able to decorate microtubules with an asymmetric tilt (Murphy and Borisy, 1975) (Figure 1A). While their consistent tilt along the length of microtubules is indicative of microtubule structural polarity, this technique never gained traction in the microtubule field for ascertaining the orientation of microtubules. Years later, however, the kinesin motor domain was used to serve a similar purpose in higher resolution EM studies (Alushin et al., 2014). Until a resolution of about 8 Å, α- and β-tubulin are structurally highly similar, making them difficult to distinguish in reconstructions to break this resolution barrier; however, decorating the microtubule with the kinesin motor domain allows tubulin dimers to be distinguished (i.e., the polarity of the microtubule is known) and allows for the identification of the microtubule seam. Thus, the kinesin motor domain served to break the symmetry of the microtubule and allowed for high resolution microtubule structures to be solved using cryo-EM.

### Analysis of supertwist of protofilaments

Analysis of moiré patterns of microtubules also suggested that microtubules were polar, as the supertwist of protofilaments had a consistent direction along the length of the microtubule, forming arrowheads (Mandelkow et al., 1986) (Figure 1B); however, establishing whether these arrowheads were pointing towards the minus- or plus-end of the microtubule was a different challenge because the handedness of this supertwist depends on the number of protofilaments (which can vary for *in vitro* polymerized microtubules) and because there is no supertwist if the microtubule has 13 protofilaments (Chrétien et al., 1996; Sosa and Chrétien, 1998).

### Hook decoration

Hook decoration, another EM-based technique, was fundamental in determining the orientation of microtubules in neurons. This technique also relies on adding a larger, more easily visible structural asymmetry to microtubules. *In vitro* work with isolated axonemes had demonstrated that, when soluble tubulin is added to microtubules in a so-called “hooking buffer,” this tubulin polymerizes into hooks along the surface of the microtubules. Importantly, because the fast- and slow-growing ends could be distinguished in these experiments, it became apparent that when viewed from the fast-growing plus-end toward the slow-growing minus-end, the hooks are right-handed (Heidemann et al., 1980; Heidemann and McIntosh, 1980). Soon after, this technique was used to study the orientation of axonal microtubules in different types of neurons, demonstrating that they are uniformly plus-end-out (Burton and Paige, 1981; Heidemann et al., 1981; Baas et al., 1988) (Figure 1D). Unlike axons, dendrites of vertebrate neurons were found to contain microtubules of mixed polarity with approximately half oriented plus-end-in and half oriented plus-end-out (Baas et al., 1988) (Figure 1F).

### Analysis of tubulin tilt in microtubule cross-sections

Interestingly, with high-resolution three-dimensional reconstructions of microtubules, the axial view of microtubules (cross-sections perpendicular to the length) also provides insight into the polarity of microtubules, as tubulin subunits are tilted counter-clockwise when looking from the plus-end towards the minus-end, and clockwise when viewed the other way around (Sosa and Chrétien, 1998) (Figure 1C). Unlike the assessment of the arrowheads present in moiré patterns due to the supertwist of protofilaments, this method is independent of the number of protofilaments. It has recently been applied in detailed cryo-electron tomography studies of axons in *D. melanogaster* and mice neurons, allowing for the assignment of microtubule polarity along with the observation of, for example, their end tapers and associated organelles (Foster et al., 2021).

### EB comet tracking

The findings on the orientation of microtubules in axons and dendrites made using hook decoration were corroborated by evidence from another technique used to assess microtubule orientation, this time based on light microscopy: end-binding (EB) comet tracking (Stepanova et al., 2003). The over-expression of fluorescently-tagged plus-end tracking proteins (+TIPs), proteins that selectively track the growing plus-ends of microtubules, allowed for the visualization of the direction of microtubule growth—and hence their orientation—in live cells [and even in mice (Kleele et al., 2014)] using light microscopy. The technical simplicity of this method allowed scientists to ascertain the orientations of microtubules in other types of neurons, including those of *D. melanogaster* and *C. elegans.* Here, unlike in vertebrate neurons, dendrites were found to contain uniformly minus-end-out microtubules (Stone et al., 2008; Hill et al., 2012). Interestingly, because observations are made with live cells, one could in theory manipulate the cells (e.g., treat them with a drug affecting the microtubule cytoskeleton) and have a read-out of the effects, if any, on microtubule organization. However, the technique does not produce images of the overall architecture of the microtubule network and only provides reliable information on the orientation of dynamic microtubules.

To bypass this limitation and also acquire orientation information about stable microtubules, EB comet tracking can be combined with laser cutting. To do so, microtubules are first severed with the hope that the newly severed microtubule ends serve as seeds for microtubule growth regardless of whether the severed microtubules were themselves dynamic or stable (Yau et al., 2016) (Figures 1E, G). However, laser severing is quite harsh on cells and might also lead to the release of Calcium ions from the endoplasmic reticulum (ER), which could in turn activate a variety of signalling pathways. Alternatively, visualizing the motility of cargoes such as Rab4-positive structures can also provide insight into how microtubules are connected between dendritic branches and, assuming that, unlike EB comets, these cargoes (also) move on stable microtubules, complement EB studies in assessing microtubule organization (Stone et al., 2008).

### Second-harmonic generation

Another technique used to visualize the parallel arrangement of microtubules in neurons that has provided insights into the organization of microtubules in brain slices of mice is second-harmonic generation (SHG) microscopy (Dombeck et al., 2003; Kwan et al., 2008). Here, trains of laser pulses are applied to live neurons or brain slices for imaging. Importantly, this technique is label-free and signal is generated *via* the structural polarity of the microtubules themselves. The signal from two adjacent parallel microtubules interferes constructively to reinforce the signal, whereas the signal from two adjacent antiparallel microtubules interferes destructively to minimize the signal. It is important to note, however, that both antiparallel and randomly mixed arrays would generate no or low SHG signal, and both 100% plus-end-out and 100% minus-end-out microtubule arrays would also generate a similar signal, restricting the use of this technique. Moreover, to interpret the signal, knowledge about the spatial distribution of microtubules (e.g., from EM studies) is required, and to put findings in context, the expression of a fluorescent marker or the subsequent staining for markers such as tau or MAP2 is also needed.

The strengths of SHG microscopy are that it works (only) with live samples and can be used to image in thick samples (i.e., brain slices). Thus, it has been used to image brain slices of mice up to 18 months old (Kwan et al., 2008). This revealed a trend in which some dendritic microtubules—specifically, in CA1 apical dendrites and the layer V cortex—become increasingly polarized within the first ∼4 months of a mouse’s life and then remained stably ∼80% polarized in older mice. In contrast, this was not observed in cultures of hippocampal neurons, in agreement with the work described above, indicating the importance of studies in brain slices and suggesting that later in life, vertebrate neurons might more closely resemble those of *D. melanogaster* or *C. elegans* with their uniform minus-end-out dendritic microtubule arrays.

### motor-PAINT

Most recently, motor-based Point Accumulation for Imaging in Nanoscale Topography (motor-PAINT), a technique simultaneously allowing for the super-resolution reconstruction of microtubules and the determination of their orientation, was developed (Tas et al., 2017). In this technique, the membranes of cells are permeabilized and the cells are gently fixed such that the structure of the microtubule cytoskeleton is preserved. Subsequently, purified fluorescent kinesin motors are allowed to explore the microtubule network and imaged for many (10,000–20,000) frames. In each frame, the particles are localized with sub-pixel precision, allowing for a super-resolution reconstruction of the microtubule network. By additionally linking the localizations of the motors in subsequent frames, the orientations of the underlying microtubules can be inferred from the directions of the tracks.

As this technique uses light microscopy rather than EM, it is easier to acquire large fields of view along the lengths of neurites and, unlike EB comet tracking, it provides information about the orientation of all microtubules regardless of dynamicity. Furthermore, the super-resolution images produced by motor-PAINT and its higher throughput nature provided insight into the ultra-structural organization of microtubules in neurites. Thus, this technique made it evident that in dendrites, microtubules are organized such that plus-end-out microtubules form bundles peripherally, while plus-end-in microtubules are bundled centrally (Tas et al., 2017) (Figures 1H, I). Moreover, this holds true over sections along the length of the dendrite (Figure 1J). One disadvantage of motor-PAINT, however, is that it requires the microtubules to be only gently fixed and accessible for kinesin binding, making it difficult to combine with other labelling or imaging strategies that would allow for the simultaneous localization of different MAPs or PTMs. However, this can be overcome by using post-fixation and re-imaging the same cell with a different super-resolution technique (Tas et al., 2017). In its current form, motor-PAINT also relies on total internal reflection fluorescence (TIRF) microscopy to minimize the effect of the high background signal due to diffusing motors, making it difficult to image throughout the cell volume or, for example, in brain slices.

What the bundled, locally-uniform organization of microtubules described above cannot explain, however, is why kinesin-1 can only drive transport into the axon, while kinesin-3 non-discriminately enters the axon and the dendrites (Lipka et al., 2016 EMBO J). To better understand this, we must also consider the diversity of microtubules present in cells, including the collection of tubulin isotypes, PTMs, and MAPs that they are associated with.

## High resolution studies of microtubule organization and diversity

In addition to their orientation, controlling microtubule architecture in cells also involves controlling the distribution of tubulin isotypes and of microtubules bearing different PTMs or MAPs. These aspects generate microtubule diversity, allowing microtubules to be specialized to fulfill different functions in cells. Sub-cellular control over the localization or enrichment of different MAPs and PTMs has been recognized for many years and is thought to be important in establishing neuronal polarity.

Early studies using diffraction-limited light microscopy revealed the asymmetric distributions of MAP2 and tau to dendrites and axons, respectively (Binder et al., 1986; Matus et al., 1986). Diffraction-limited microscopy can thus reveal large scale distinctions in the distribution of a MAP or PTM, such as its localization to a given neurite, whether it is enriched proximally or distally along a neurite, or whether it is, for example, enriched near branch points. Early work from cells with radial microtubule arrays, however, already made it apparent that adjacent microtubules can “look” very different, with studies indicating that microtubules are not uniformly decorated with PTMs, but that these modifications tend to segregate to specific microtubules, as seen with acetylation (Thompson et al., 1984) and detyrosination (Gundersen et al., 1984). However, getting a better understanding of the role and distribution of different types of microtubules in neurons with their densely packed axonal and dendritic microtubule arrays [spacing of ∼20–70 nm (Chen et al., 1992)], requires microscopy techniques with better resolution.

This is an interesting question to consider because neurons critically depend on microtubule-based transport, and the kinesin and dynein motors driving this transport have been shown to preferentially use microtubules decorated by a specific PTM and be activated or inhibited by the presence of different MAPs. Thus, the tubulin code and MAP code are likely important regulators of microtubule-based transport in neurons; however, the precise roles of many MAPs and PTMs remain largely unresolved.

### Single-molecule localization microscopy

**FIGURE 2:**
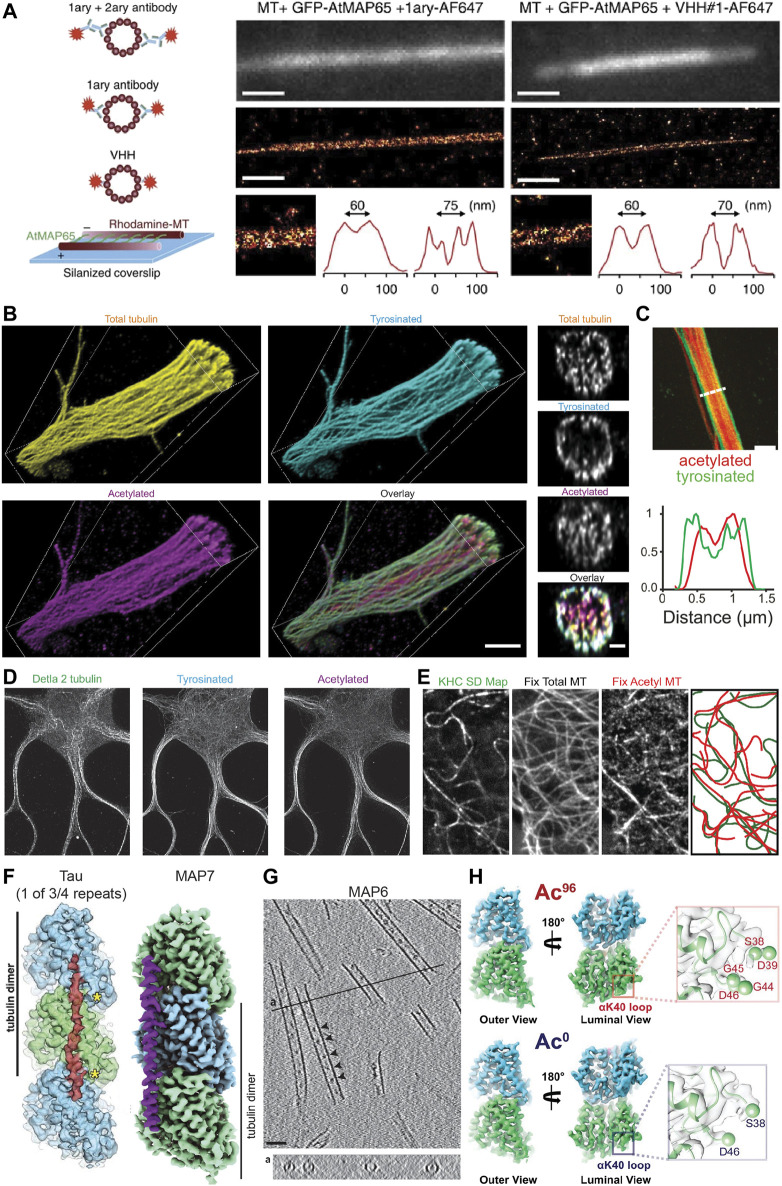
Methods for visualizing aspects of the MAP code and the tubulin code. **(A)** An illustration showing different labelling strategies [primary + labelled secondary, labelled primary, and labelled nanobody (here VHH)] used in single molecule localization microscopy approaches such as STORM to highlight their effect on the effective microtubule diameter. Experimental set-up to create microtubules cross-linked with a known distance of 30–40 nm between microtubules or 55–65 nm between microtubule centers using MAP65/Ase1 from *A. thaliana*. An experimental comparison of the observed peak-to-peak distances for microtubules crosslinked with MAP65 when imaged using a labelled primary antibody (left) or a labelled nanobody (right). Note that microtubules can be more easily distinguished with the nanobody. Taken from (Mikhaylova et al., 2015). **(B)** Volumetric 3D rendering of microtubules in a dendrite as imaged with four-fold expansion microscopy, as well as corresponding cross-sections. Shown are total tubulin, tyrosinated tubulin, acetylated tubulin, and an overlay. Note that acetylated microtubules are enriched centrally, while tyrosinated microtubules are enriched peripherally. Taken from (Katrukha et al., 2021). **(C)** A Stimulated Emission Depletion (STED) microscope image of a dendrite from a DIV7 rat hippocampal neuron similarly showing the central and peripheral enrichment of acetylated and tyrosinated microtubules, respectively. This is also visualized in the line scan below. Note, however, that the resolution here is much worse than with expansion microscopy. Taken from (Tas et al., 2017). **(D)** STED image of a DIV9 rat hippocampal neuron stained for Δ2, tyrosinated, and acetylated tubulin. Interestingly, Δ2 tubulin is also enriched centrally in dendrites, perhaps more tightly than acetylated tubulin. Taken from (Katrukha et al., 2021). **(E)** A correlative experiment in which kinesin-1 (KHC [1–560]-3xmCitrine) motility was imaged in live cells (observed tracks shown as a standard deviation map) and cells were subsequently fixed and stained for total tubulin and acetylated tubulin. The microtubules utilized by kinesin-1 are predominantly acetylated. Taken from (Cai et al., 2009). **(F)** The footprint of the microtubule-binding domain of tau and the structure of MAP7 as solved by cryo-EM. Tau binds at the very ridge of the protofilament, whereas MAP7 binds away from this site towards the lateral contacts. Depending on the isoform, tau has either three or four microtubule-binding pseudo-repeats that bind along a single protofilament, only one of which is shown. As each microtubule-binding domain binds across an inter-dimer interface, adjacent tubulin dimers can be stapled together by the microtubule-binding repeats. The microtubule binding region of MAP7 similarly spans across an inter-dimer and an intra-dimer interface, but the protein does not stretch as far along a protofilament. Taken from (Kellogg et al., 2018) and (Ferro et al., 2022). **(G)** Cryo-electron tomography of *in vitro* microtubules polymerized from purified tubulin in the presence of GMPCPP and MAP6, showing that MAP6 forms particles (indicated by arrowheads) at regularly spaced intervals inside the microtubule lumen (also shown in the cross-section below). Taken from (Cuveillier et al., 2020). **(H)** Maps of ∼96% acetylated and < 1% acetylated microtubules as solved by cryo-EM. Note that the overall structures of the tubulin dimer look very similar regardless of acetylation status. However, changes can be observed in the loop in the lumen of the microtubule on which αK40 is found in that the loop becomes more ordered upon acetylation. This can be seen here in that more residues appear in the reconstruction of the acetylated tubulin dimer, thereby suggesting that they are less flexible after acetylation. Taken from (Eshun-Wilson et al., 2019).

Understanding the track selectivity of motor proteins and how PTMs and MAPs regulate neuronal transport requires us to visualize how different types of microtubules are organized in these cells. To resolve the precise organization of microtubules, super-resolution techniques are needed, especially when studying bundled or dense microtubule arrays, such as are found in neuronal processes. Multiple studies have used PAINT or Stochastic Optical Reconstruction Microscopy (STORM) approaches to achieve this. In general, these techniques do not allow for individual microtubules within bundles to be resolved as the linkage error resulting from the physical size of the primary and secondary antibodies increases the effective microtubule diameter by ∼25 nm, such that the closely bundled microtubules in dendrites are blurred together (Figure 2A). One way to overcome this is to use labelled nanobodies, which can reduce the linkage error from ∼12.5 to ∼2.5 nm (Mikhaylova et al., 2015) (Figure 2A). Thus, one could imagine generating multiple nanobodies to simultaneously resolve different types of PTMs or MAPs in neurons.

Individual microtubules have also been distinguished in bundles using an approach similar to motor-PAINT (Balabanian et al., 2017), and motor-PAINT has recently been combined with MINimal photon FLUXes (MINFLUX) microscopy (see section “*Live-cell markers and microscopy*” below) as the improved resolution of this technique allows microtubules to be distinguished more easily (Deguchi et al., 2023). While this method does not allow different types of microtubules (e.g., those decorated by different PTMs) to be distinguished directly (as kinesin-1 selectivity is not preserved *in vitro*), it allows single microtubules and bundles to be distinguished and can be combined with subsequent antibody stainings and STORM to visualize different PTMs. This can provide novel insights, such as the preferential bundling of acetylated microtubules in COS-7 cells (Balabanian et al., 2017), and when looking at microtubule orientation, that plus-end-in microtubules, which are bundled centrally in dendrites, are predominantly acetylated, while peripheral plus-end-out microtubules are enriched in tyrosinated tubulin (Tas et al., 2017). These correlative findings suggests that, while microtubules in dendrites might be of mixed orientation, local uniformity in microtubule type and orientation through bundling could create “super-highways” for microtubule-based transport.

### Expansion microscopy

Another increasingly popular method that greatly improves the effective resolution in light microscopy is expansion microscopy (ExM) (Chen et al., 2015; Tillberg et al., 2016). Recent improvements in the method have allowed for up to ten-fold expansion (Damstra et al., 2022; Li et al., 2022; Klimas et al., 2023) and near single-microtubule resolution in dendrites even with four-fold expansion using Flip-ExM, facilitating a precise mapping of how acetylated and tyrosinated microtubules are organized in dendrites, including quantifying the absolute numbers of microtubules (Jurriens et al., 2021; Katrukha et al., 2021) (Figure 2B). As seen previously with STED (Figure 2C) and STORM (Tas et al., 2017), this study confirmed with improved (3D) resolution that in dendrites, acetylated microtubules are bundled centrally, Δ2 tubulin is enriched centrally (Figure 2D), and tyrosinated microtubules are located peripherally. Moreover, this work found that most microtubules are accounted for when considering acetylated and tyrosinated microtubules (Katrukha et al., 2021), and because other PTMs are also found in dendrites, this—not surprisingly—indicates that microtubules must bear multiple PTMs. Thus, while acetylation is often considered a marker for stable microtubules, perhaps acetylated and Δ2-modified microtubules are even more stable. It is unclear, however, how many acetylated microtubules are Δ2-modified and *vice versa*. Resolving single microtubules will be important to categorize them into different groups based on one or more properties.

One of the key advantages of ExM over many other super-resolution techniques is that the effective resolution is improved not only in *x* and *y*, but also in *z*. Thus, it should be possible to determine, for example, whether MAPs preferentially localize to a specific subset of microtubules, are enriched on one side of a microtubule or if they are located inside a microtubule rather than on its surface. Protofilament-specific MAP and microtubule inner protein (MIP) localization has been seen on ciliary doublet microtubules using cryo-EM (Ma et al., 2019), and it will be interesting to see if this protofilament-specific localization of MAPs and/or MIPs can also be seen for neuronal microtubules to define highways for different types of cargoes. Getting such precise information with ExM may require, for example, the use of nanobodies and/or post-expansion labelling to reduce the linkage error, which is also expanded (Tillberg et al., 2016; Zwettler et al., 2020). Thus, with typical primary and secondary antibody stainings using Ten-fold robust expansion (TREx) microscopy (Damstra et al., 2022), the linkage error of 12.5 nm on either side of the microtubule (Mikhaylova et al., 2015) is expanded to about 125 nm, so the microtubule’s observed diameter is double its true diameter. When using nanobodies, which result in a smaller linkage error of about 2.5 nm (Mikhaylova et al., 2015), the linkage error would be 6x less. If post-expansion labelling is used, the linkage error would not be expanded, decreasing its contribution to the observed microtubule diameter ten-fold such that the linkage error would contribute < 10% of the observed microtubule diameter.

### Cryo-electron microscopy

Determining precisely how MAPs bind the microtubule or observing structural changes in the tubulin dimer, however, requires the structural context and resolution provided by EM. Improvements in the technique and the analysis methods have allowed scientists to solve the structure of tubulin within the microtubule under various conditions, as well as the structure of MAPs decorating the microtubule lattice, with impressive detail. For example, the structure of Protein Regulator of Cytokinesis 1 (PRC1), tau, and MAP7 bound to the microtubule have been solved with near atomic resolution, with PRC1 binding at the intradimer interface, tau binding along the ridge of a protofilament and linking multiple subsequent tubulin dimers, and MAP7 binding along a protofilament, but extending less far along it and binding more towards the inter-protofilament contact sites rather than at the ridge (Kellogg et al., 2016; Kellogg et al., 2018; Ferro et al., 2022) (Figure 2F). This detailed structural information provides insight into how these proteins function. For example, one can precisely compare the binding footprint of these proteins with that of kinesin or dynein to better understand how these proteins might interact on the microtubule surface. Such analysis has been done for MAP4, suggesting that it can bind microtubules without impeding the binding of kinesin-1 (Shigematsu et al., 2018). In another study, a combination of EM techniques was used to study MAP6 function, revealing that this MAP is actually a MIP as it localizes to the inside of microtubules both *in vitro* and in neurons, that it can coil microtubules, and that it introduces apertures along the microtubule lattice (Cuveillier et al., 2020) (Figure 2G).

In addition to revealing these precise interactions, EM can also provide information about the microtubule lattice under different conditions. It is known that tubulin can adopt both an expanded (∼8.4 nm) and a compacted (∼8.2 nm) form at least in part depending on its bound nucleotide, with GTP-bound tubulin being more expanded than GDP-bound tubulin (see also “*Defining stable microtubules*” below) (Alushin et al., 2014; LaFrance et al., 2022). Interestingly, however, it appears that certain MAPs, such as the neuronal MAPs tau and MAP2, can induce local compaction of the lattice at points where they bind cooperatively to form so-called islands or envelopes (Siahaan et al., 2022). In contrast, kinesin-1 has been shown to expand the microtubule lattice, at least *in vitro*, with ∼10% lattice occupancy (Peet et al., 2018; Shima et al., 2018). Furthermore, a kinesin-1 rigor (with a mutation that prevents it from hydrolyzing ATP, rendering it non-motile) has been shown to localize preferentially to microtubules with an expanded lattice in cells (de Jager et al., 2022). Thus, there is some flexibility in the structure of tubulin and this could serve as a means for proteins to bind cooperatively and communicate allosterically *via* the microtubule lattice (Brouhard and Rice, 2018). Indeed, studying how these proteins interact to induce lattice compaction or expansion has been the focus of some recent work (Siahaan et al., 2022) and it will be interesting to see if this differs, for example, between dendrites and axons, near synapses or branch points, and on different microtubule subsets.

In theory, PTMs could also impact tubulin structure; however, most occur on the flexible C-terminal tails of α- or β-tubulin, making it less obvious how local structural changes, if any, could be propagated to the rest of the tubulin dimer. Despite this, the C-terminal tails themselves adopt a variety of transient conformations and this “structural landscape” could be altered by PTMs such as polyglutamylation. This could in turn influence how proteins interact with the microtubule (Bigman and Levy, 2020; Chen et al., 2021). One commonly-studied PTM that occurs on the body of the tubulin dimer, albeit in the lumen of the microtubule, is the acetylation of αK40 and the effects of this PTM on the structure of tubulin have been investigated; however, it appears that the acetylation of this residue does not lead to large-scale allosteric structural changes in the tubulin dimer that could be easily recognized by proteins binding to the outside of the microtubule. Instead, it leads to more subtle changes in the lateral interactions between tubulin dimers (Howes et al., 2013; Eshun-Wilson et al., 2019) (Figure 2H), which could still be detected, e.g., by some MIPs or MAPs that bind between protofilaments.

### Correlative live and fixed cell approaches

Despite these advances in imaging methods and insights into how MAPs and PTMs influence microtubule structure, comprehensive studies allowing us to query the precise sub-cellular distribution of these features as was done for the distribution of acetylated and tyrosinated microtubules in dendrites (Katrukha et al., 2021) or understand the functional significance of their structure and localization have been limited. This is due in part to the fact that most of the techniques discussed above do not allow for live-cell imaging, which would be beneficial to assess microtubule-related functions (e.g., cargo motility). For example, it is unclear whether different dendritic cargoes preferentially use either the central highways of plus-end-in acetylated microtubules or the peripheral highways of plus-end-out tyrosinated microtubules.

To bypass this, some correlative work has been done in which transport is observed in live cells, which are subsequently fixed and the same cell is imaged by a super-resolution technique such as STORM to assess how cargoes traverse microtubule crossings (Bálint et al., 2013) or to better trace microtubules along their lengths and assess certain characteristics such as damage sites (Hao et al., 2020). Correlative studies with diffraction limited light microscopy were also done to demonstrate that kinesin-1 preferentially moves on acetylated microtubules *in vivo* (Reed et al., 2006; Cai et al., 2009) (Figure 2E). Furthermore, such correlative studies also revealed that lysosomes are enriched on detyrosinated microtubules, and by knocking down motors of the kinesin-1 (KIF5B), kinesin-2 (KIF3A), and kinesin-3 (KIF1B) family, it was established that KIF5B is responsible for this enrichment on detyrosinated microtubules (Mohan et al., 2018). This suggests that this motor preferentially moves on detyrosinated microtubules [which are also usually acetylated (Katrukha et al., 2021)] even when bound to endogenous cargo that is simultaneously bound to other motors. However, most of this work has been done in cell culture lines with a radial microtubule array rather than in neurons, as the density of the microtubule cytoskeleton in neurons makes it difficult to establish precisely which microtubule a cargo was moving on.

### Live-cell markers and microscopy

Because the microtubule cytoskeleton is constantly rearranging, it would be beneficial to develop markers to visualize microtubule diversity in live cells. To this end, CRISPR-based approaches (Jinek et al., 2012; Doudna and Charpentier, 2014; Willems et al., 2020; Droogers et al., 2022) will be important to determine the endogenous distribution of MAPs and tubulin isotypes, but this cannot be used to visualize PTMs or whether microtubules are in an expanded or compacted state. One approach to generate a live cell marker for a given PTM is to screen a library of mutants for a binder that specifically associates with, e.g., tyrosinated microtubules and then express a fluorescently-tagged version thereof in cells (Cassimeris et al., 2013; Kesarwani et al., 2020). One could also imagine a more targeted approach by engineering fluorescent probes based on proteins or protein domains that have been observed to specifically associate with given PTMs, e.g., the CAP-Gly domain which has been shown to associate with tyrosinated tubulin (Peris et al., 2006) or, for example, engineering mutant enzymes [e.g., αTAT1 or a Tubulin Tyrosine Ligase Like (TTLL) family member] that specifically associate with deacetylated or non-glutamylated tubulin but do not themselves alter the PTM status of tubulin. These live cell markers, however, would have limited utility in neurons when trying to investigate the precise partitioning of these modifications within neurites unless combined with super-resolution imaging techniques that allow for near single-microtubule resolution. Most of these techniques (e.g., STORM and PAINT) are incompatible with live cell imaging because they often require special buffers or high laser powers that are damaging to cells and/or have poor time resolution. One technique that is promising for live-cell super-resolution microscopy is MINFLUX (Gwosch et al., 2020; Schmidt et al., 2021). Here, an oscillating donut-shaped excitation beam and a confocal pinhole detector are used to calculate the position of a given dye molecule with ∼1–3 nm resolution. Currently, however, this technique is limited to single wavelengths, small fields of view, and is slow because it iteratively localizes or tracks each dye molecule in turn. Despite this, it has been recently used to visualize the stepping of kinesin-1 both *in vitro* (Wolff et al., 2022) and *in vivo* (Deguchi et al., 2023), demonstrating its promise for visualizing dynamics with incredible resolution.

## Studying microtubule dynamics and stability

These and other live-cell imaging techniques will also aid the study of another hallmark property of microtubules: their dynamic instability. This ability to rapidly transition between growth and shrinkage allows them to quickly reorganize into drastically different architectures, as is beautifully exemplified with the mitotic spindle or during neurite outgrowth or axonal pruning. However, microtubules do not all have the same dynamic behaviour. Early on, the micro-injection of tubulin into fibroblast cells allowed scientists to assess the turnover rate of microtubules, revealing that most microtubules have a half-life of ∼10 min, while a smaller sub-population has a half-life of ∼1 h (Schulze and Kirschner, 1987). Thus, the existence of a stable subset of microtubules was identified in some cell lines. Furthermore, it seemed as though these were two distinct subsets given that the micro-injected tubulin either fully labelled microtubules along their lengths or was completely absent from other microtubules. This was also the case after some hours, although the number of unlabelled microtubules decreased with time, suggesting that they do turnover at longer timescales (Schulze and Kirschner, 1986; Schulze and Kirschner, 1987). These stable microtubules were further described to be clustered around the center of the cell and curly in comparison to the rather straight dynamic microtubules (Schulze and Kirschner, 1987). Despite the early discovery of these stable microtubules, two matters complicated their study, especially in neurons: first, there is also evidence that these are not two distinct populations of microtubules, but rather that many microtubules have a stable base and a dynamic plus-end (Baas and Black, 1990; Baas and Ahmad, 1992; Ahmad et al., 1993; Baas et al., 2016; Qiang et al., 2018); and second, the tools to study the behaviour of this rather small subset of microtubules, particularly in live cells, have been limited. This is because these stable microtubules are largely found in areas where the microtubule density is highest (e.g., near the MTOC in cells with a radial microtubule array and in axons and dendrites); because their slow turnover rate makes them difficult to label *via* pulse-chase type methods; and because they are massively outnumbered by dynamic microtubules in most cell types studied, making them difficult to visualize.

### Studying microtubule dynamics *in vivo*

Studying the dynamics of dynamic microtubules, however, has been successful both *in vitro* and *in vivo*. To study their polymerization *in vivo*, EB comet tracking can be used. Here, the overexpression of EB1 or EB3 at low levels allows one to visualize the growing plus-ends of microtubules by the selective association of EBs immediately behind the GTP cap of growing microtubules (Stepanova et al., 2003; LaFrance et al., 2022) (Figures 1E, G). Tracking these EB comets allows you to assess the growth rates of microtubules, the frequency of growth events, the orientation of dynamic microtubules, and even estimate more detailed parameters such as the rate of GTP hydrolysis based on the comet decay length (Duellberg et al., 2016; Roostalu et al., 2020). EBs, however, also influence microtubule dynamics by promoting microtubule growth, while simultaneously increasing the catastrophe frequency (Maurer et al., 2012; Maurer et al., 2014). One way to minimize this effect is to express an SxIP-motif-containing protein, which then localizes to the plus-ends by binding to EB1, rather than expressing EBs directly (Yau et al., 2016). This construct may in turn, however, compete with endogenous EB binding partners.

### Studying microtubule dynamics *in vitro*

**FIGURE 3:**
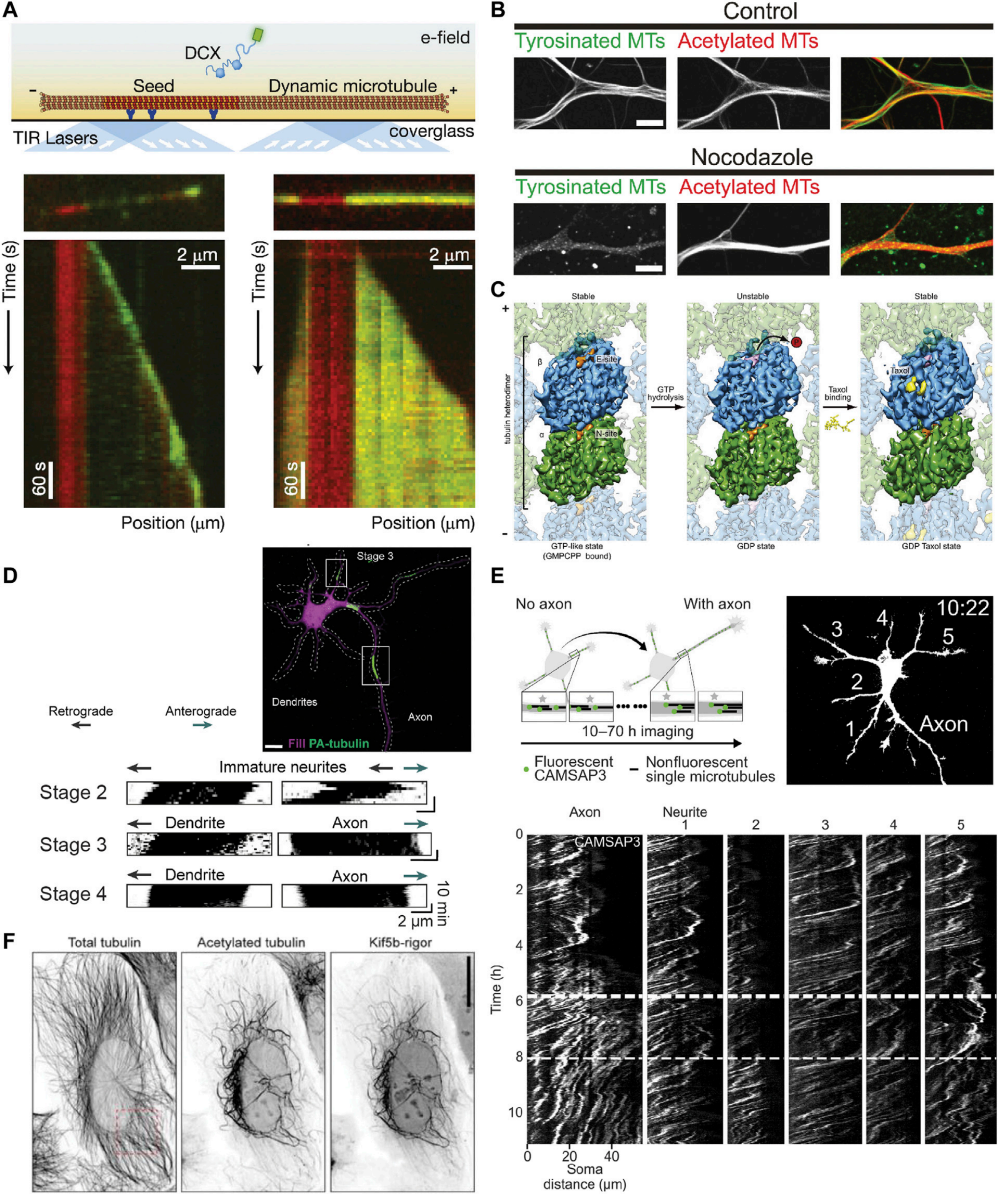
Methods for studying the properties of stable microtubules. **(A)** Schematic showing an *in vitro* assay to study microtubule dynamics and how it is affected by different MAPs. A microtubule seed stabilized with GMPCPP is immobilized *via* antibodies/streptavidin on an otherwise passivated coverslip and soluble tubulin (and in this case DCX) are added with GTP, such that the tubulin can undergo cycles of nucleated growth and shrinkage. Below are kymographs of two microtubules with the microtubule seed visible in red, and DCX visible in green at two different concentrations: 10 nM (left) and 100 nM (right). Interestingly, DCX shows different behaviours at these two concentrations. Taken from (Bechstedt and Brouhard, 2012). **(B)** Image showing tyrosinated and acetylated tubulin stainings in DIV9 rat hippocampal neurons in control conditions (left) or after a 2.5 h treatment with 4 µM nocodazole. Note how without the nocodazole treatment, the overall levels of the tyrosinated and acetylated microtubules is similar, but the low dose nocodazole treatment preferentially depolymerizes dynamic (tyrosinated) microtubules such that very little tyrosinated microtubule signal is present on the right, while the level of acetylated microtubules is less affected. Taken from (Tas et al., 2017). **(C)** Cryo-EM maps of tubulin within the microtubule lattice (viewed from the lumen) with tubulin bound to GMPCPP (a GTP analog) (left), GDP (middle), or GDP + Taxol (right). Note that overall, the GMPCPP-bound tubulin and Taxol-stabilized tubulin look similar, having a longer lattice spacing (expanded conformation), while GDP-bound tubulin is more compacted. As indicated, both GMPCPP- and Taxol-bound microtubules are stable, while GDP-bound microtubules are not, suggesting that lattice expansion and microtubule stability are intricately connected. Taken from (Alushin et al., 2014). **(D)** A stage 3 (axon specified) rat hippocampal neuron expressing mRFP-fill (magenta) and photoactivatable-tubulin (green) with converted regions also indicated. The motion of the photoactivated tubulin can be clearly tracked for ∼10 min, as shown in the kymographs underneath for neurites in stage 2, 3, and 4 neurons. This reveals the predominantly retrograde motion of microtubules in neurites except for the one designated as an axon. Taken from (Burute et al., 2022). **(E)** Experimental set-up and image of mCherry-fill in a stage 2-to-3 mouse hippocampal neuron expressing mCherry-fill and mNeonGreen-CAMSAP3. CAMSAP3-labelled microtubules could be tracked for much longer periods of time (10–70 h) than photoactivated-tubulin, as evidenced by the kymographs showing the faster retrograde flow in all neurites except the axon once specified. Taken from (Schelski and Bradke, 2022). **(F)** U2OS cells expressing KIF5B-rigor tagged with 2xmNeonGreen fixed and stained for acetylated and total tubulin. Note how the KIF5B-rigor preferentially decorates the subset of microtubules that are acetylated, a commonly used marker for stable microtubules. Taken from (Jansen et al., 2023).

To more precisely study microtubule dynamics and how they are influenced, for example, by different MAPs, *in vitro* assays in which templated microtubule growth is observed using TIRF microscopy are commonly performed. Here, short, stabilized microtubule segments (“microtubule seeds”) are attached to a coverslip *via* an antibody or streptavidin within a flow chamber. Soluble tubulin is then introduced, which, in the presence of GTP, can polymerize onto the ends of these microtubule seeds, producing microtubules that undergo cycles of growth and shrinkage (Gell et al., 2010) (Figure 3A). These assays have given rise to our stereotypical picture of microtubule dynamic instability and have allowed us to understand how a wide variety of proteins or other factors influence microtubule dynamics and *vice versa*. For example, DCX tip tracks at lower concentrations and in this way helps to ensure that microtubules have 13 protofilaments (Bechstedt and Brouhard, 2012). Such *in vitro* assays have also contributed to our understanding of kinesin-3 track selectivity, as this motor was demonstrated to preferentially dissociate near the plus-ends of microtubules regardless of initial binding site, and to rarely start runs in this region (Guedes-Dias et al., 2019). This suggests that kinesin-3 prefers to bind to and move along the GDP-tubulin lattices found along the microtubule shaft rather than the GTP-tubulin found in the GTP cap. Because microtubule plus-ends are enriched at *en passant* boutons in rat hippocampal neurons, this was suggested to improve cargo delivery at these pre-synaptic sites (Guedes-Dias et al., 2019).

### Defining stable microtubules based on their lifetime and structure

If these dynamic microtubules are the stereotypical microtubules that we picture when we think about microtubules, then what exactly are stable microtubules and what makes them stable? We will here use the working definition that stable microtubules are just that: stable. This should be the case when cells are exposed to cold temperatures or treated with low doses of nocodazole (a drug that binds to tubulin and interferes with microtubule polymerization), as has been observed for a subset of microtubules in different types of cells including neurons (Figure 3B), where they are, in dendrites, mostly oriented plus-end-in (Schulze et al., 1987; Tas et al., 2017). Treatment with higher concentrations of calcium ions can also be used to destabilize microtubules, but not all cold-stable microtubules appear to be resistant to calcium treatment—at least in the optic nerve of three-month-old rats (Song et al., 2013). This suggests that there might be different degrees of microtubule stability.

When we think of stability in terms of microtubules, we often think of the stable GTP cap. Based on work with GTP analogs and hydrolysis-deficient mutants (Alushin et al., 2014; LaFrance et al., 2022), it has been suggested that tubulin dimers bound to GTP have an expanded lattice compared to GDP-tubulin (dimer rise of ∼8.4 nm instead of ∼8.2 nm). Similarly, Taxol-stabilized microtubules also resemble GTP microtubules in that they are expanded, albeit in a slightly different manner (Alushin et al., 2014) (Figure 3C). *In vitro*, microtubules can also be stabilized by expanding their lattice using Taxol or the slowly hydrolysable GTP analog GMPCPP. Because no additional factors are required, this suggests that lattice expansion is directly responsible for stabilizing the microtubule lattice. Earlier work suggested that lateral contacts are largely unaltered by the expansion/compaction occurring at the interdimer interface and that instead, longitudinal contacts along the protofilament are responsible for microtubule stability (Alushin et al., 2014); however, more recent work has suggested that lattice compaction strengthens longitudinal interactions and weakens lateral interactions, which thus rupture first when a microtubule transitions to a catastrophe (Manka and Moores, 2018; LaFrance et al., 2022). The compaction around the nucleotide binding site in β-tubulin upon GTP hydrolysis is thus thought to introduce strain into the lattice of dynamic microtubules, promoting rapid depolymerization upon loss of the GTP cap (Alushin et al., 2014; Manka and Moores, 2018).

### Identifying factors that could impart stability on microtubules

Lattice expansion or, conversely, lattice compaction upon GTP hydrolysis, is likely also detectable by other proteins, especially those that bind at the interdimer interface (Alushin et al., 2014). One protein that binds at this site is EB3 (Zhang et al., 2015b), the commonly-used marker for microtubule dynamics that binds near the growing plus-end. Interestingly, EBs do not only recognize, but promote lattice compaction (LaFrance et al., 2022), suggesting that they help growing microtubules quickly transition to a compacted state [as is associated with GDP-tubulin (Alushin et al., 2014)] and that dynamic microtubules are compacted along their length.

Furthermore, if an expanded lattice is what renders a microtubule stable, then lattice expansion must be induced (or lattice compaction prevented) to generate and maintain stable microtubules in the cell, for example, by different MAPs recruited to specific microtubules. One candidate for doing so could be MAP6, which has been shown to render microtubules resistant to depolymerization upon drug or cold treatment (Bosc et al., 2003; Delphin et al., 2012; Cuveillier et al., 2020). Indeed, MAP6 appears to form particles inside microtubules (Figure 2G) and has been suggested to resist lattice compaction after GTP hydrolysis, resulting in coiled MTs, at least *in vitro* (Cuveillier et al., 2020). It will be interesting to see if it is found in all stable microtubules or if there are other microtubule stabilizers capable of expanding the microtubule lattice. For example, structural MAPs such as tau and MAP2 have been suggested to stabilize microtubules; however, it is unclear if this is a true stabilization of the microtubule lattice given that they compact rather than expand the microtubule lattice (Siahaan et al., 2022). Instead, they might “stabilize” the lattice by limiting its dynamicity, providing additional interactions that must be broken in order to depolymerize the microtubule or by bundling microtubules to limit how accessible they are to severing enzymes; this would not necessarily render them resistant to cold-dependent or nocodazole-induced depolymerization. Thus, which MAPs can impart stability on a microtubule and how is something that requires further investigation.

It is, of course, also possible that PTMs help keep microtubules in an expanded state. One candidate PTM here could be polyamination. Cold-stable microtubules have been shown to be enriched in polyaminated tubulin, particularly in neurons (Song et al., 2013). Interestingly, one of the major sites of amination appears to be Glutamine 15 on β-tubulin (βQ15), placing a positively charged amine group near the GTP-binding site of β-tubulin (Song et al., 2013). This could serve to stabilize the negatively charged phosphate groups of GTP to limit hydrolysis or otherwise minimize the conformational changes resulting from hydrolysis to keep polyaminated microtubules stable during their renewal. Many other PTMs are at sites where it would be difficult to imagine how they directly stabilize microtubules; however, they could promote stabilization (or dynamicity) by recruiting specific MAPs (Chen et al., 2021).

Another idea that has recently garnered a lot of interest is that motor proteins might stabilize the microtubule tracks they use most by promoting the exchange of tubulin along the length of the lattice such that there are more GTP-tubulin islands (i.e., small patches of microtubule rich in GTP-tubulin) peppered throughout the microtubule (Théry and Blanchoin, 2021; Triclin et al., 2021; Andreu-Carbó et al., 2022); however, the extent to which wildtype and motile motors do this might be limited (Budaitis et al., 2021). The idea is that the GTP islands along the length of the microtubule might help to protect the microtubule from catastrophe by serving as rescue sites to prevent complete catastrophes (Vemu et al., 2018; Bollinger et al., 2020). Conceptually, this is distinct from the idea of stability being a property of some microtubules along their length as it would mean that (large sections of) microtubules are not uniformly stable and instead that microtubules are resistant to depolymerization at these GTP-tubulin-rich sites, but otherwise undergo cycles of growth and (limited) shrinkage. This idea, however, suggests that stable microtubules incorporate fresh GTP-tubulin throughout the shaft, which does not agree with previous observations that stable microtubules have limited subunit turnover along their lattice (Schulze and Kirschner, 1987).

One way to reconcile the idea of patchy microtubules and limited dynamicity with microtubules being stable along their length is if the GTP-tubulin present in the islands allosterically induces an expanded conformation in neighbouring GDP-tubulin dimers; however, the neighbouring GDP-tubulin dimers could also allosterically induce their compacted conformation in the GTP-tubulin dimers, which may further speed up the hydrolysis of GTP such that the lifetime of GTP-tubulin within the lattice (even in these islands) is very brief. While these are possibilities, it is unclear how well allosteric changes can be propagated longitudinally along the microtubule lattice given that for kinesin-1 to induce the expansion of a whole a microtubule, ∼10% lattice occupancy is required (more than 1 kinesin per cross-sectional “ring” of tubulin) (Peet et al., 2018) and given that tau and MAP2 can induce lattice compaction that is not propagated along the length of the lattice (Siahaan et al., 2022).

### Examining the relationship between acetylation, detyrosination, and stability

Various attempts have been made to study the dynamics and behaviour of stable microtubules. Early studies used antibody blocking techniques in which cells are incubated with biotin-tubulin that gets incorporated into dynamic microtubules, which can then be blocked using streptavidin. Stable microtubules, which did not incorporate biotin-tubulin remain accessible for antibody binding during subsequent stainings for PTMs on these stable microtubules, specifically acetylation. This work found that stable microtubules are typically acetylated (Schulze et al., 1987). Indeed, acetylation is often used as a marker for stable microtubules, but this does not mean that no dynamic microtubules are acetylated or that acetylation directly promotes stability. Similarly, detyrosination, another marker for stable microtubules, is likely not directly responsible for stability (Khawaja et al., 1988; Webster et al., 1990); however, it can affect interactions with MAPs and motors such as Cytoplasmic Linker Protein (CLIP) 170/EB1 (Chen et al., 2021) and the depolymerizing mitotic centromere-associate kinesin (MCAK) (Peris et al., 2009), thereby affecting microtubule dynamics indirectly. This might also help explain why detyrosinated microtubules seem unable to serve as templates for fresh microtubule growth from their (plus-)ends (Schulze and Kirschner, 1986; Webster et al., 1987; Infante et al., 2000; Palazzo et al., 2001).

Interestingly, the amount of nocodazole-resistant (i.e., stable) microtubules in cells was described to be reduced in αTAT1-depleted RPE cells (Xu et al., 2017) and increased in Tubulin Tyrosine Ligase (TTL)-depleted neurons (Peris et al., 2009). These studies suggest that these modifications can (indirectly or directly) affect microtubule dynamics or stability. Indeed, it was shown *in vitro* that acetylated microtubules are more resistant to mechanical stresses due to an increase in the flexibility of their lattices *via* reduced lateral interactions between protofilaments (Portran et al., 2017; Xu et al., 2017). Furthermore, some localized changes in tubulin structure have been observed upon acetylation (Figure 2H), but it is difficult to imagine how these could directly influence microtubule stability because of the limited changes observed in the (longitudinal) contacts upon acetylation (Howes et al., 2013; Eshun-Wilson et al., 2019).

While Taxol stabilizes microtubules *in vitro* without inducing acetylation, when it is added to cells, microtubules are rapidly acetylated (Piperno et al., 1987; Xiao et al., 2006) and also show an increased level of detyrosination (Gundersen et al., 1987). This suggests that, in general, acetylation and detyrosination are indeed markers for stable microtubules and the increase in acetylation and detyrosination upon Taxol addition could be because Taxol stabilizes (i.e., increases the lifetime) of all microtubules, allowing sufficient time for the accumulation of these PTMs. Alternatively, it could also indicate that microtubule stabilization *via* lattice expansion promotes the acetylation and/or detyrosination of microtubules (Zhang et al., 2003), for example, by enhancing the binding or activity of the tubulin acetyltransferase αTAT1 or of one of the detyrosinating enzymes vasohibin (VASH)1/VASH2-small vasohibin binding protein (SVBP) (Nieuwenhuis et al., 2017) or microtubule-associated tyrosine carboxypeptidase (MATCAP) (Landskron et al., 2022).

### Tracking bulk movements and sliding of microtubules

To study the behaviour of a population of microtubules, the photoconversion or photoactivation of tubulin can also be used. Here, tubulin is activated in a given region and the signal from dynamic microtubules dissipates due to depolymerization whereas the signal from stable microtubules remains, allowing the motility of these microtubules to be observed (Jolly et al., 2010; Burute et al., 2022) (Figure 3D). This method does not allow for long-term imaging, as the signal from stable microtubules also disperses over longer periods of time and the fluorophores relax back to their original state. Thus, long-term imaging, as was needed to visualize the retrograde flow of microtubules in neurites during the earlier stages of neuronal development required a different approach: the use of CAMSAP3 as a minus-end marker (Schelski and Bradke, 2022) (Figure 3E). However, it is unclear which minus-ends CAMSAP3 labels when over-expressed, so this cannot currently be used to specifically say something about the behaviour of stable microtubules.

### Visualizing stable microtubules in live cells

Recently, a new tool has emerged to study stable microtubules, enabling their long-term tracking: a fluorescent rigor kinesin-1 construct (G234A) that cannot effectively hydrolyze ATP (Rice et al., 1999) and selectively binds to stable microtubules in cells: Stable Microtubule-Associated Rigor Kinesin (StableMARK) (Jansen et al., 2023) (Figure 3F). The stable microtubules marked by this construct, which are largely acetylated and detyrosinated, were observed to slide and curl in cells. Moreover, in agreement with the idea that stable microtubules are stable along the length of their lattice rather than being stabilized *via* caps at either extremity, rigor-decorated microtubules rarely depolymerized when they were laser-cut to generate newly exposed plus- and minus-ends. Interestingly, the rigor was observed to transiently associate with many microtubules in the cell, but had a high unbinding-rate from dynamic microtubules such that it only accumulated on stable microtubules. This marker was also shown to preferentially associate with (stable) microtubules with an expanded lattice in U2OS cells (de Jager et al., 2022) and *in vitro* (Jansen et al., 2023). This makes it interesting to consider how this compares with the track selectivity of wild-type (motile) kinesin-1.

As KIF21B (in the kinesin-4 family) also preferentially binds to non-dynamic, stable microtubules to move with a retrograde bias in dendrites (Masucci et al., 2021), it will also be exciting to see if these motors recognize the same features of stable microtubules. Alternatively, these motors could recognize very different features, both of which are associated with stable microtubules. For example, one motor could directly recognize the conformation of tubulin within stable microtubules, while another could indirectly do so *via* a PTM or MAP that recognizes stable microtubules or induces microtubule stability.

## Understanding the formation and function of distinct microtubule subsets

This brings us to the question: what kinds of subsets might there be in neurons and what might the roles of these subsets be? As discussed above, cells appear to have dynamic microtubules with a half-life on the order of minutes (∼10 min in fibroblast cells) and stable microtubules with a half-life on the order of many minutes to hours (∼1 h in fibroblast cells) (Schulze and Kirschner, 1987). When we think about microtubules, we typically think of dynamic microtubules due to their well-studied dynamic instability and thus their importance can be easily appreciated; being highly dynamic allows the microtubule cytoskeleton to quickly rearrange during cell division or in response to signalling cues, as well as to facilitate cell motility or form branches. While the importance of stable microtubules might be less apparent, acknowledging another major role of microtubules—namely, to serve as tracks for transport—makes it evident that having some form of continuity in these tracks, as could be facilitated by stable microtubules, would be beneficial. These stable microtubules can also provide structural support for cells and, as we will see below, might be involved in reinforcing key decisions taken during neuronal development. In this section, we will thus begin to explore the properties of these subsets in terms of their signature of MAPs and PTMs, how they are built and maintained in neurons, and what roles they play in the cell.

### Characteristics of dynamic microtubules

Without the addition of MAPs or drugs, microtubules are dynamic *in vitro*. Similarly, as dynamic microtubules outnumber stable microtubules in cells, it seems as though being dynamic is the default state of microtubules *in vivo*. These microtubules are marked by a compacted, GDP-tubulin lattice and undergo cycles of growth and shrinkage. During growth phases, they bear a cap of GTP-tubulin and have EB comets and other +TIPs at their growing end. EBs help to compact tubulin within the lattice (LaFrance et al., 2022; Zhang et al., 2018b), suggesting that all but the most distal tubulin dimers are in the compacted conformation. This conformation likely renders these microtubules less resistant to treatment with low doses of nocodazole. Indeed, microtubules marked by non-acetylated and tyrosinated tubulin largely disappear after this treatment in dendrites (Tas et al., 2017). Given the short lifetime of dynamic microtubules, they are likely composed largely of such unmodified tubulin. These microtubules are further found peripherally in dendrites and largely oriented with their plus-ends distally (Tas et al., 2017).

The compacted conformation of dynamic microtubules is preferred and promoted by some MAPs, including tau and MAP2 (Siahaan et al., 2022), which thus likely associate primarily with these dynamic microtubules; however, it remains to be determined if these MAPs are also enriched peripherally in dendrites, where dynamic microtubules are found. Furthermore, structural changes in the microtubule lattice could promote or limit motor binding. For example, as kinesin-1 has been suggested to prefer and induce an expanded GTP-like lattice (Nakata et al., 2011; Peet et al., 2018; Shima et al., 2018), MAPs that induce lattice compaction could inhibit kinesin-1 from moving effectively along these compacted microtubules by limiting kinesin-1 binding.

Many of the MAPs enriched on these microtubules, such as DCX, DCLK1, tau, and MAP2, have been shown to have inhibitory effects on kinesin-1 (Lipka et al., 2016; Monroy et al., 2020), perhaps also helping to explain why kinesin-1 appears to avoid these microtubules in cells. It will be interesting to see if MAP9 and SEPT9, which also inhibit kinesin-1 (Karasmanis et al., 2018; Monroy et al., 2020), preferentially bind to dynamic microtubules. Interestingly, some of these same MAPs are permissive to or even promote kinesin-3 motility, including DCX, DCLK1, SEPT9, and MAP9 (Karasmanis et al., 2018; Monroy et al., 2020). This might help to explain why kinesin-3 appears to prefer dynamic, tyrosinated microtubules in cells (Tas et al., 2017), in addition to its apparent preference for GDP-tubulin lattices (Guedes-Dias et al., 2019).

However, some MAPs that are likely on dynamic microtubules, namely, tau and MAP2, do inhibit kinesin-3 motility (Monroy et al., 2020). This suggests that these MAPs may be on a subset of dynamic microtubules or on a different part of the lattice than kinesin-3. Interestingly, although tau is often said to stabilize microtubules, this “stability” seems to result from tau promoting longer labile ends of microtubules rather than stabilizing the microtubule as a whole (Qiang et al., 2018), suggesting that it may indeed be found on dynamic microtubules, but towards the growing ends. In line with this, tau has also been shown to limit the formation of EB comets in cells (Ramirez-Rios et al., 2016). As EB comets are found at the ends of dynamic microtubules, this suggests that tau may similarly be found on these microtubules and perhaps even near the (plus-)ends, where processive runs by kinesin-3 are generally not observed (Guedes-Dias et al., 2019).

### Characteristics of stable microtubules

While dynamic instability is an intrinsic property of microtubules, stability seems to be something that has to be imparted on them. We hypothesize that the hallmark of these microtubules, marked in cells by extensive PTMs including acetylation, polyamination, detyrosination, Δ2-modification, methylation, polyglutamylation, and/or polyglycylation (depending on cell type and intracellular location), is their expanded lattice (see “*Defining stable microtubules*” above). This expanded conformation likely directly imparts stability along the length of these microtubules and renders them resistant to both growth and shrinkage such that they have a comparatively long lifetime (∼1 h).

MAPs associated with stable microtubules likely prefer and/or promote this expanded state. It will be interesting to identify which MAPs are capable of modulating microtubule stability and if they do so by being present during microtubule polymerization or afterwards. For example, MAP6, which is known to stabilize microtubules, must be present during microtubule polymerization to fulfill its role, perhaps because it is localized inside the microtubule lumen or because it acts at the growing tip of the microtubule (Cuveillier et al., 2020).

Allosteric conformational changes in the microtubule lattice could additionally help keep certain MAPs off of stable microtubules, thereby facilitating kinesin-1-based transport. For example, tau and MAP7 compete for binding on the microtubule lattice (Monroy et al., 2018), but do not directly share a binding site (Kellogg et al., 2018; Ferro et al., 2022). If MAP7 were to promote an expanded tubulin conformation unfavourable for tau binding, this could help displace tau from the lattice. Indeed, MAP7 is able to out-compete tau *in vitro* (Monroy et al., 2018), but it is unclear whether this occurs *via* a modulation of the lattice conformation. In this way, MAPs could define “highways” for different motor proteins by decorating distinct microtubules and having divergent effects on these motor proteins. Thus, (stable) microtubules decorated by MAP7 would promote kinesin-1 transport, while (dynamic) microtubules decorated by tau would inhibit it (Dixit et al., 2008; Barlan et al., 2013; Hooikaas et al., 2019). Given that tau inhibits kinesin-1 motility even at low concentrations and that kinesin-1 prefers stable microtubules, tau must either be (almost) absent from stable microtubules or there must be different types of stable microtubules of which kinesin-1 utilizes those with MAP7 but lacking tau. However, more studies will be needed to ascertain whether MAP7 preferentially decorates stable microtubules and if it is found on all stable microtubules or a subset of them. For example, is MAP7 enriched centrally in dendrites, where stable, plus-end-in microtubules are found (Tas et al., 2017; Katrukha et al., 2021)? Tau also appears to compete with MAP6 in axons, with tau allowing microtubules to have longer, labile ends, while MAP6 produces stable (non-dynamic), cold-resistant microtubules (Qiang et al., 2018) (see also “*Identifying factors that could impart stability on microtubules*”). Given that MAP6 forms particles within the microtubule lumen, it certainly does not share a binding site with tau on the microtubule lattice, suggesting that allosteric effects could be at play. As MAP6 is found in (some) stable microtubules, this could further help ensure that tau does not bind along the length of these microtubules, again helping to define potential “highways” for kinesin-1.

Such MAPs might additionally recruit a given set of tubulin modifying enzymes or, by inducing changes in tubulin conformation, could render a given residue more accessible to a given tubulin modifying enzyme. This is an especially attractive idea for PTMs occurring on the body of tubulin or those catalyzed by enzymes that bind the tubulin dimer in a manner that would be sensitive to lattice expansion and compaction. For example, the expansion of the microtubule lattice may give αTAT1 better access to the loop on which αK40 is found or the detyrosinating enzyme MATCAP, which binds along the microtubule ridge near the interdimer interface (Landskron et al., 2022), could be well positioned to read out changes in lattice conformation and may bind better to expanded lattices. Alternatively, given that the activity of HDAC6 has not been tested on microtubules with a compacted lattice and that it shows very little activity on microtubules with an expanded lattice i.e., stabilized with Taxol or GMPCPP (Skultetyova et al., 2017), it could theoretically be that lattice expansion dramatically reduces the affinity/activity of HDAC6 [in line with a previous suggestion that Taxol somehow reduces the activity of HDAC6 (Xiao et al., 2006)] or SIRT2. Thus, the conformation or stability of the microtubule lattice and the MAPs decorating it could be coordinated with the PTMs that it bears.

### Roles of microtubule subsets

**FIGURE 4:**
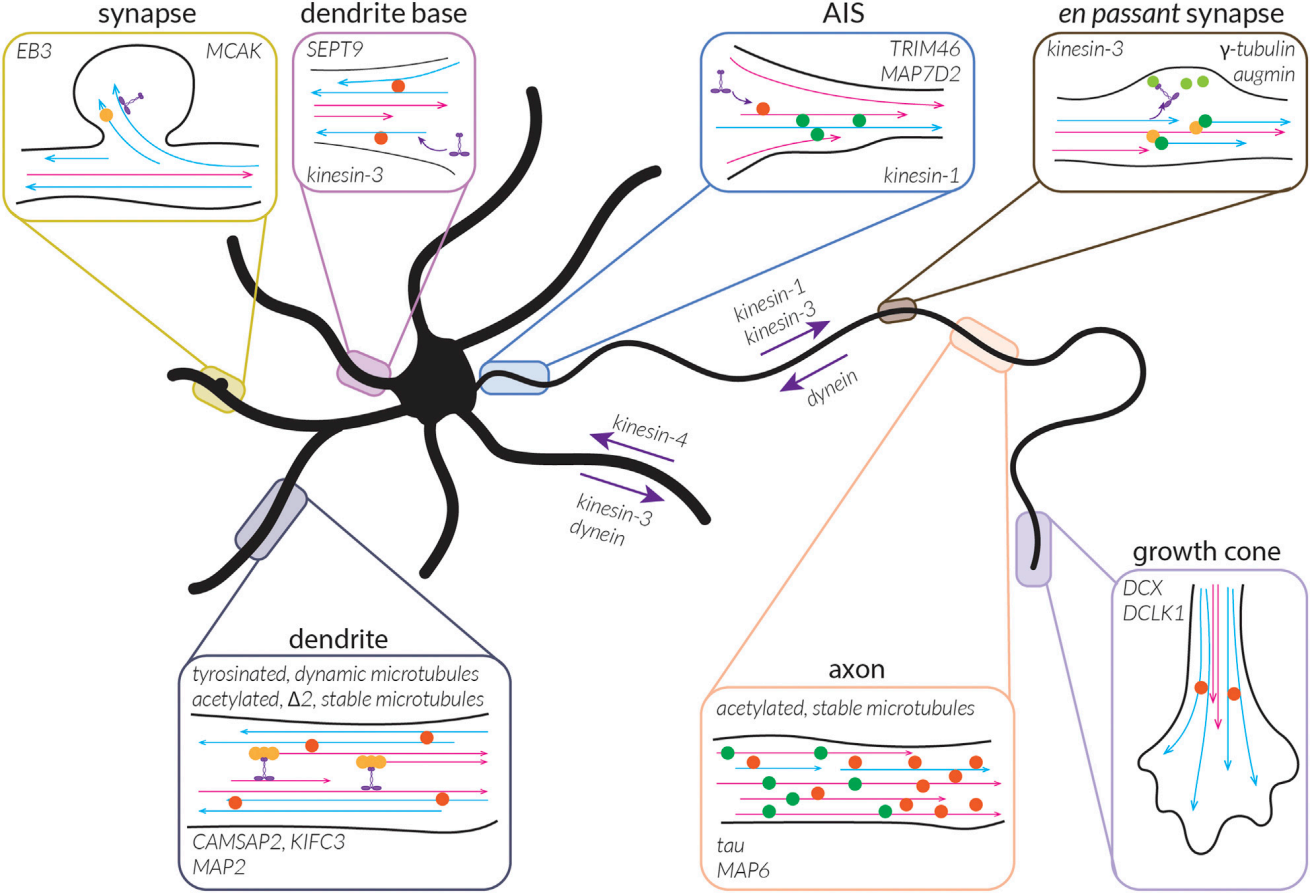
A map of the neuronal microtubule cytoskeleton and associated factors. The neuronal microtubule cytoskeleton is organized into a spectacularly asymmetric array composed of two main subsets of microtubules: stable and dynamic (indicated by magenta and cyan lines, respectively). Their stability/dynamicity, motility, organization, and nucleation are controlled by a variety of MAPs, some of which are shown here at the site within the neuron where they are believed to serve an important role. The subdivision of the microtubule cytoskeleton into these subsets, along with their respective organization (and orientation), likely helps to control the transport of different cargoes by motor proteins, which thus serve as anterograde or retrograde motors in different neuronal processes. Some of these (including their direction of transport within an axon/dendrite) are indicated. Note that for some of these MAPs, it is not known whether they bind specifically to stable or dynamic microtubules. The localization of tubulin modifying enzymes is not known, so these are not depicted.

Microtubules have many important roles in neurons thereby necessitating that the microtubule cytoskeleton be organized by a slew of MAPs that locally regulate microtubule nucleation, dynamics, organization, and motility to generate and maintain populations of dynamic and stable microtubules (Figure 4). The resulting microtubule subsets are important to direct intracellular transport by different members of the kinesin family and dynein into or out of neurites. To query this selectivity, one can use peroxisome redistribution (PEX) assays in which a peroxisomal membrane-targeting signal is fused with 12-kDa FK506 binding protein (FKBP12), a domain that can bind to the FKBP-rapamycin binding domain (FRB) in the presence of rapalog (Kapitein et al., 2010b). This FRB domain is fused to a motor protein of interest such that, after the addition of rapalog, the motor is linked to peroxisomes and redistributes them from their usual perinuclear location according to the traffic rules that dictate that motor’s movement in the cell. In this way, it has been shown that KIF5A,B,C (kinesin-1), KIF3A and KIF17 (kinesin-2), KIF13A,B and KIF16B (kinesin-3), KIF4A,B (kinesin-4), KIF11 (kinesin-5), KIF20A,B and KIF23 (kinesin-6), KIF18A,B (kinesin-8), KIF22 (kinesin-10), and KIF15 (kinesin-12) selectively move peroxisomes into the axon, while KIF1A,B,C (kinesin-3) and KIF21A,B (kinesin-4) move peroxisomes into both the axon and the dendrites (Lipka et al., 2016), albeit with a retrograde bias for KIF21B in the dendrites (Masucci et al., 2021). However, this does not mean that these axon-specific motors are not found in dendrites; rather, it suggests that these motors do not move anterogradely in dendrites, but they could still act as retrograde motors in these processes using the plus-end-in microtubules. Indeed, this is precisely what has been described for KIF17 (kinesin-2) (Kapitein et al., 2010b; Franker et al., 2016). It will thus be a topic for future studies to see how this selectivity is orchestrated in cells and which MAPs or PTMs help guide these motor proteins and their associated cargo to the right destination.

Stable microtubules might be especially important to allow for proper organelle positioning and to provide continuity for transport, especially by kinesin-1. Kinesin-1 has indeed been shown to prefer acetylated (i.e., stable) microtubules in cells (Reed et al., 2006; Cai et al., 2009; Tas et al., 2017), whereas it is indifferent to this modification *in vitro* (Walter et al., 2012; Kaul et al., 2014). This indicates that kinesin-1 is not recognizing the PTM directly, but rather some other aspect of stable microtubules. This selectivity holds true also for kinesin-1-driven cargoes. For example, ER tubules are positioned in part by kinesin-1, and reticulon-4 (RTN4; a protein helping to shape ER tubules) has been shown to co-localize with acetylated (i.e., stable) microtubules (Farías et al., 2019), in agreement with the microtubule preference of kinesin-1. ER tubule distribution was also impacted by Taxol, which stabilizes microtubules, and higher doses of nocodazole, a drug that depolymerizes microtubules. Furthermore, ER-mitochondrial contacts have also been shown to occur on cold-resistant and acetylated microtubules in COS-7 cells (Friedman et al., 2010), and lysosomes are enriched on detyrosinated microtubules as a result of KIF5B function (Mohan et al., 2018).

Kinesin-3, while closely related to kinesin-1, has very different preferences for microtubules. Indeed, it appears to bind more strongly to dynamic microtubules and GDP lattices (Tas et al., 2017; Guedes-Dias et al., 2019). While it is unclear which aspect of kinesin-1 and/or kinesin-3 is responsible for this difference in track selectivity, one well-established difference in the motor domains of kinesin-1 and kinesin-3 is the presence of the K-loop (multiple positively-charge Lysine residues in loop 12 in the motor domain) on kinesin-3. The K-loop of kinesin-3 has been swapped into kinesin-1, and this mutant kinesin-1 is able to enter dendrites and axons instead of being axon-specific like wild-type kinesin-1 (Huang and Banker, 2012). How can the presence of the K-loop in the motor domain of kinesin-1 override its axon specificity? It appears that this change could be due, not to a change in tubulin PTM preference, but rather to a change in regulation by a MAP: SEPT9. This MAP is found predominantly somatodendritically in neurons and was shown to inhibit kinesin-1 but promote kinesin-3 motility, keeping kinesin-1 out of the dendrites while allowing kinesin-3 to enter; kinesin-1 with kinesin-3’s K-loop is no longer inhibited by SEPT9, allowing it to enter dendrites (Karasmanis et al., 2018). In contrast, MAP7D2 is thought to help drive kinesin-1 based transport into the axon (Pan et al., 2019).

Dynein, on the other hand, has been found to initiate motility more on tyrosinated microtubules not *via* a preference of the motor itself, but rather *via* preferential binding to these microtubules by the CAP-gly domain of a component of its cofactor dynactin, p150^Glued^ (Peris et al., 2006; McKenney et al., 2016). Whether the selectivity of other motors for a given PTM is also mediated *via* cofactors or adaptor proteins remains an interesting question. For kinesin-1, one might imagine that MAP7 fulfills this role as MAP7 recruits and activates kinesin-1 both in cells and *in vitro* (Hooikaas et al., 2019; Monroy et al., 2020). Interestingly, MAP7D1 appears to localize to some subset of microtubules in HeLa cells (Hooikaas et al., 2019), but what these microtubules are and if this is the case in neurons is unclear. Unlike kinesin-1 and kinesin-3, dynein motility is largely unaffected by most MAPs except for MAP9 (Monroy et al., 2020); however, as it is the main minus-end-directed transport motor in our cells, it is logical that dynein should be able to walk on a variety of microtubules. MAPs such as MAP9 could then help dictate whether dynein serves as an anterograde or retrograde motor in a given region e.g., in dendrites (Kapitein et al., 2010a).

Given the segregation of stable and dynamic microtubules in dendrites, it will also be interesting to see how microtubule subsets impact the transport and distribution of cargo such as mitochondria, lysosomes, and endosomes, particularly when these cargoes are carried by multiple motors with differences in their preferred microtubule tracks. For example, dynamic microtubules serve as tracks for KIF1A (kinesin-3) and dynein, whereas stable microtubules serve as tracks for KIF5A,B,C (kinesin-1) and KIF21B (kinesin-4). What happens if a cargo is bound by both kinesin-1 and kinesin-3: can it be carried into dendrites and, if so, does it continue to move outwards by the action of kinesin-3 on the dynamic plus-end-out peripheral microtubules? Are there other cargoes that preferentially move inwards on the stable plus-end-in central microtubules through the action of kinesin-1?

In addition to their roles in serving as tracks for transport by some motor proteins, dynamic microtubules are important in processes in which the cell must migrate, change shape or respond to signalling cues. For example, when neurons alter their dendritic spines, as might occur during synaptic plasticity, dynamic microtubules penetrate these spines, helping to regulate the actin network that is primarily responsible for upholding their structure (Jaworski et al., 2009; Uchida and Shumyatsky, 2015). These microtubules may in part be regulated by DCLKs, which localize to distal dendrites and help promote dendrite outgrowth and limit synapse maturation (Shin et al., 2013). Furthermore, it has been shown that the re-tyrosination of tubulin by TTL is important for the invasion of dendritic spines by dynamic microtubules, as the reduction of TTL, which reduces the level of tyrosinated tubulin also leads to dendritic spine loss in mice (Peris et al., 2022). Detyrosinated microtubules may less readily enter spines due to their limited recruitment of CLIP170/EB1, which can give tyrosinated microtubules a higher growth rate and catastrophe frequency (Chen et al., 2021), or their limited recruitment of MCAK (Peris et al., 2009), which has also been shown to have important roles in dendritic spines (Zheng et al., 2022). These dynamic, tyrosinated microtubules preferentially entering dendritic spines could additionally serve as tracks for transport by, e.g., kinesin-3 or dynein, both of which prefer these microtubules (McKenney et al., 2016; Tas et al., 2017; Guedes-Dias et al., 2019). The presence of dynamic microtubules at *en passant* boutons (in axons) has also been described to allow for kinesin-3-mediated cargo delivery at these presynaptic sites (Guedes-Dias et al., 2019). This would imply the localization of GTP-rich plus-ends of microtubules to these *en passant* synapses. However, it has also been observed that microtubule nucleation often initiates at these boutons *via* the localized action of γ-tubulin and augmin, suggesting that microtubule minus-ends are also enriched at these sites (Qu et al., 2019). Interestingly, this phenomenon is activity-dependent and local microtubule nucleation increases with treatments that induce neural activation or action potential firing. Abrogation of this local microtubule nucleation impaired synaptic vesicle delivery. Thus, microtubule dynamics play important roles both post- and pre-synaptically.

More dramatically, microtubules are also involved in neuronal development (including neurite outgrowth) and migration, which many neurons undergo to move from their site of cell division to their final position (Lasser et al., 2018). For example, microtubules have been implicated in neurite extension and their presence and roles in growth cones and axon pathfinding have been well-documented (Challacombe et al., 1997; Sánchez-Huertas and Herrera, 2021; Atherton et al., 2022). Many of these microtubules in growth cones are dynamic and mice lacking TTL (i.e., with lower levels of tyrosinated tubulin) display abnormalities in terms of neurite outgrowth (Erck et al., 2005). Indeed, proteins that regulate microtubule nucleation and dynamics play important roles in growth cones. For example, DCX, which can nucleate 13-protofilament microtubules (Bechstedt and Brouhard, 2012), is enriched in growth cones (Tint et al., 2009) and MAP1B, which regulates microtubule dynamics likely by regulating the binding of EBs (Tortosa et al., 2013), also has important roles in axonal outgrowth and branching (Tymanskyj et al., 2012; Kiss et al., 2018). While a role for dynamic microtubules in growth cones is rather obvious, some microtubules in this region are stable. These stable microtubules might serve to reinforce growth or other decisions made during development. One example of this is seen during the transition from stage 2 to stage 3 of neuronal development, when local microtubule stabilization is sufficient to dictate axon formation. This was demonstrated through the use of photoactivatable Taxol, which allowed microtubules to be selectively stabilized in one neurite, promoting that neurite to become the axon (Witte et al., 2008). In addition, a global Taxol treatment induces the formation of multiple axons (Hammond et al., 2009). Thus, it appears as though microtubule stabilization plays an important role in axon specification and perhaps more generally, in decision making. However, recent work has shown that in the spinal projection neurons of zebrafish, microtubules may not be completely necessary for axon specification *per se*, but rather for the stabilization and dilation of the emerging axonal protrusions (Moore et al., 2022).

### Maintaining distinct subsets within neurites: Microtubule orientation

**FIGURE 5:**
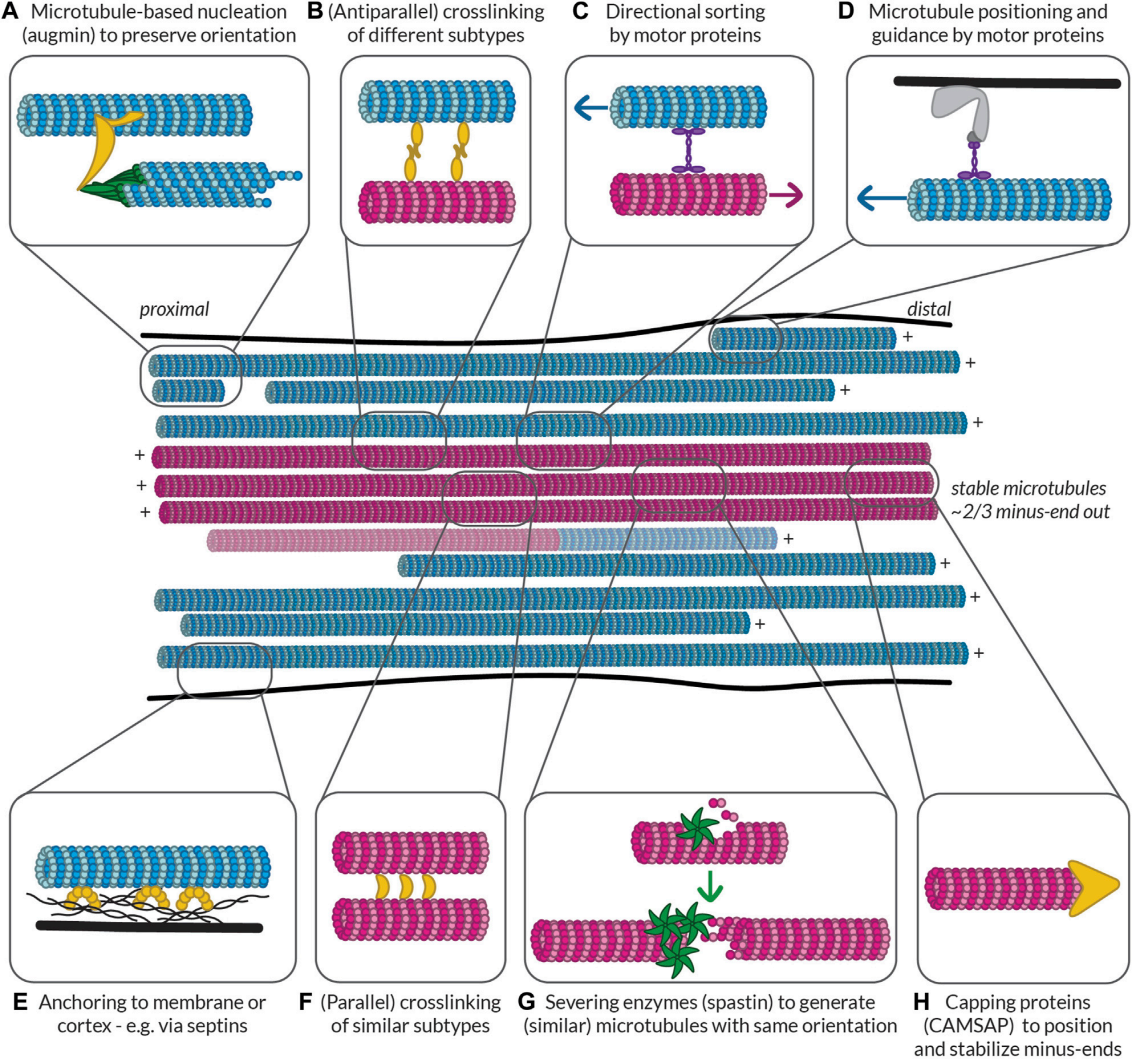
Possible ways to build and maintain (partially) segregated dendritic microtubule subsets. Some of the mechanisms that could be at play to help establish and maintain subset identity in terms of orientation, PTMs, and MAP decoration. Controlled nucleation, such as with **(A)** augmin and **(G)** off of microtubule seeds generated by spastin-mediated severing can help preserve local microtubule orientation. Different mechanisms can then help **(F)** bundle similar microtubules *via* (parallel) crosslinkers, **(B)** link different bundles *via* (antiparallel) crosslinkers, and **(C)** keep uniform orientation within bundles by pushing out microtubules of the opposite orientation. Microtubules can also be properly positioned based on their orientation, PTMs, or MAPs *via* **(D)** motor proteins anchored to the membrane or cortex or **(E)** other MAPs such as septins anchored to the membrane or cortex. Finally, **(H)** capping proteins such as CAMSAPs can help to position the (minus-)ends of microtubules properly relative to, e.g., branch points or synapses. All these proteins could recognize the relative orientation of microtubules or the complement of MAPs and PTMs that they bear. Furthermore, by bundling similar microtubules, tubulin-modifying enzymes and MAPs could move between microtubules to help maintain subset identity. Magenta microtubules are stable (i.e., have an expanded lattice and are decorated with the associated MAPs such as MAP6 and PTMs such as acetylation and detyrosination), while blue microtubules are dynamic (i.e., have a compacted lattice and are decorated with MAPs such as EB and are tyrosinated). There is a possibility that some microtubules are mixed and have a stable base and a dynamic end, so such a microtubule is also shown.

With these subtypes co-existing in close proximity in cells, particularly in dendrites, we must also consider how their distinct identities can be established and maintained (Figure 5). As neurons progress through the initial stages of development to form neurites that grow and shrink as well as specify an axon, the centrosome is still active (Yu et al., 1993). Subsequently, however, the centrosome is inactivated, indicating that dendritic outgrowth depends entirely on alternate nucleation strategies (Stiess et al., 2010; Cunha-Ferreira et al., 2018). Given that nucleation likely plays an important role in maintaining the proper organization of the microtubule cytoskeleton including the orientation of microtubules and their identity, mapping out microtubule minus-ends and identifying nucleation sites will be an important topic for future studies. Indeed, once nucleated, microtubules likely have a fixed orientation within the dendrites and axon, as the limited diameter of neurites and how crowded they are would likely prevent microtubules from being rotated. Thus, microtubule orientation would be dictated by how microtubules are nucleated, and various methods have been proposed for directionally-biased nucleation to establish and maintain the proper orientation of microtubules in dendrites and axons.

In particular, much effort has been invested in identifying nucleation sites and mechanisms for polarity sorting in *D. melanogaster* and *C. elegans* neurons following the inactivation of their centrosome. These include alternate MTOCs such as γ-tubulin and Rab11-positive organelles in the dendritic growth cones of *C. elegans* that are localized by kinesin-1 (Liang et al., 2020); nucleation from Golgi outposts in dendrites (Ori-McKenney et al., 2012) or from somatic Golgi compartments with kinesin-2 guiding plus-end-out microtubules into the axon (Mukherjee et al., 2020) in *D. melanogaster* neurons; guiding and sorting of microtubules by motor proteins such as kinesin-2 (Mattie et al., 2010), kinesin-5 (Feng et al., 2021), and dynein (Rao et al., 2017); branching nucleation off of existing microtubules (Sánchez-Huertas et al., 2016; Cunha-Ferreira et al., 2018; Thawani et al., 2019); and microtubule severing to create new microtubule seeds (McNally et al., 2006; Srayko et al., 2006; Kuo and Howard, 2021).

While some of these alternative MTOCs could be present in vertebrate (i.e., human) neurons, evidence to this end is limited. Furthermore, in the dendrites of vertebrate neurons there is the additional challenge of building and maintaining a proper ratio of plus-end-in to plus-end-out microtubules that is coordinated with differences in microtubule stability and PTMs. Moreover, the cell must preserve the local uniformity of microtubule subtypes in dendrites with plus-end-in, stable, acetylated microtubules concentrated centrally and plus-end-out, dynamic, tyrosinated microtubules enriched peripherally. This is in contrast to *C. Elegans* or *D. melanogaster* neurons, which both have uniformly minus-end-out microtubules in their dendrites.

One way that microtubule orientation could be locally preserved in the dendrites of vertebrate neurons is through branched microtubule-based nucleation using the γ-tubulin ring complex (γTuRC) associated with the side of an existing microtubule *via* the human Augmin complex (HAUS) (Sánchez-Huertas et al., 2016; Cunha-Ferreira et al., 2018) (Figure 5A). In axons it has additionally been shown that such γ-tubulin- and Augmin-dependent nucleation can be regulated by neuronal activity (Qu et al., 2019). Whether this is the case in dendrites remains to be determined. Similarly, microtubule severing (by spastin) (Vemu et al., 2018; Kuo et al., 2019; Kuo and Howard, 2021) and the use of the severed microtubules as templates for new growth is an attractive mechanism to facilitate this (Figure 5G). Additionally, motor sliding and orientation-dependent microtubule crosslinking e.g., by Ase1/MAP65 which crosslinks antiparallel microtubules with a fixed spacing (Gaillard et al., 2008) or by TRIM46 which bundles parallel microtubules in the axon initial segment (Van Beuningen et al., 2015) could be important to form bundles of uniform polarity and ensure that no microtubules pointing the “wrong” way are incorporated into a bundle of the opposite polarity (Figures 5B–D, F).

### Maintaining distinct subsets in neurites: MAPs and PTMs

These mechanisms would serve to establish a neuronal dendritic microtubule network with balanced microtubule orientation, but how can distinct subsets in terms of MAP binding, PTM decoration, and microtubule stability be maintained? For example, if there is a dominant MAP, what prevents it from binding to every microtubule and restricts it to a given subset of microtubules? Similarly, how are PTMs limited to only some of the microtubules in a given dendrite? One way to achieve this is if most tubulin modifying enzymes and MAPs have a very low basal activity or affinity and are activated or recruited by another specific MAP or tubulin conformation. If this is the case and most MAPs and modifying enzymes are unable to bind and act on newly nucleated microtubules, how are microtubules able to associate with any MAPs or tubulin modifying enzymes?

This is reminiscent of a problem with chromatin and defining active and silent chromatin; here, pioneer transcription factors that enable the binding of other transcription factors are the first to bind and often modify the chromatin in such a way that it becomes more favourable for binding (Zaret and Carroll, 2011). Similarly, there could be pioneer MAPs or modifying enzymes that alter the microtubule structure to allow other proteins to bind. Once a microtubule is designated as stable by such pioneering MAPs or enzymes, other MAPs or enzymes could begin to act. Perhaps they can only bind cooperatively and their initial binding has a large energetic barrier, but once one is bound, the energetic barrier for other (similar) MAPs or enzymes to bind is lowered. For example, for PTMs on the C-terminal tail of tubulin such as polyglycylation or polyglutamylation, the modifying enzymes could bind better to already modified C-terminal tails. This would allow the enzymes to also modify the tails of neighbouring tubulin dimers, thereby creating a positive feedback loop such that microtubules are more uniformly modified. In addition to such feedback loops, this interplay between writers and readers of the tubulin code (Yu et al., 2015) could allow one modification to recruit enzymes to modify the microtubule in a different way. These “writers that read” are again similar to what has been proposed for the histone code and the maintenance of histone modifications (Zhang et al., 2015c). Indeed, the parallels between the histone code and the tubulin code might be even more direct than proposed here, as some of the same enzymes (e.g., SETD2, HDAC6) appear to be involved in modifying both (Liu et al., 2012; Park et al., 2016).

Such feedback loops might help cells to preserve subset identity for newly nucleated microtubules when severed microtubules are used to template new growth; if the base of a daughter microtubule is decorated by a certain set of MAPs and PTMs, the same set of MAPs and PTMs could be recruited to the newer part of the microtubule. This could occur, for example, by the modified tubulin dimers in the seed serving as a preferred binding site for specific tubulin modifying enzymes. The MAPs present could also move along the lattice *via* diffusion and/or recruit a certain set of MAPs or exclude others either *via* competitive binding or through lattice allostery—i.e., converting the microtubule lattice into a (non-)favourable conformation for binding. The interplay between these features would allow the cell to define microtubule subsets in terms of their orientation, stability or dynamicity, MAP-decoration, and PTMs.

Furthermore, microtubules decorated with a certain MAP or PTM could also selectively be crosslinked to each other or anchored to the plasma membrane or actin cortex to allow similar microtubules to be bundled and properly localized within a dendrite (Figures 5D, E). This could occur along the length of the microtubule or, for example, at the minus-ends of the microtubule by CAMSAP2, which has been demonstrated to be important for axon specification and dendritic outgrowth (Yau et al., 2014) (Figure 5H). This would ensure that minus-ends marked by CAMSAP2 are appropriately localized within the dendrite, for example, in relation to synapses. By selectively bundling and positioning microtubules oriented parallel to one another, MAPs or tubulin modifying enzymes could move between adjacent microtubules to locally maintain a subset. In this way, subset identity could also be imparted on new, locally nucleated microtubules. What remains unclear, however, is how tubulin isotypes fit into this picture.

## Discussion

As microtubules are required for both neuronal development and function, it is easy to guess that many microtubule-related proteins are mutated in neurodevelopmental and neurodegenerative disorders (as listed, e.g., in (Maday et al., 2014; Bonini et al., 2017; Varidaki et al., 2018)). These include mutations in genes corresponding to tubulin isotypes (e.g., α-tubulin 1a, β-tubulin 2b), MAPs (e.g., tau, DCX, spastin), motor proteins (e.g., KIF1A, KIF21A), cargo adaptors (e.g., huntingtin, Lis1), and tubulin modifying enzymes [e.g., TTLL11, cytosolic carboxypeptidase 1 (CCP1) (Magiera et al., 2018; Shashi et al., 2018)]. In turn, one might imagine that the microtubule cytoskeleton or its associated proteins could function as effective drug targets to slow or reverse the progression of these diseases.

Indeed, this has been tested with the use of microtubule stabilizing agents such as taxanes to promote axon regeneration after spinal cord injury (Hellal et al., 2011) and to counteract neurodegeneration in cellular or mouse models (Cartelli et al., 2013; Fernandez-Valenzuela et al., 2020) of Alzheimer’s or Parkinson’s disease. Synthetic stabilizers have also been generated and used in cellular and mouse models of tauopathy (Kovalevich et al., 2016; Zhang et al., 2018a). As these are microtubule stabilizers, this could indicate that the balance of stable and dynamic microtubules might be shifted towards too many dynamic microtubules in neurodegenerative disorders and that these drugs help to restore the balance. However, any drugs reaching clinical trials have so far proven to be inefficacious (Tsai et al., 2020; Boiarska and Passarella, 2021). In fact, whereas some studies suggested that there were lower levels of acetylated tubulin in the brains of patients with Alzheimer’s disease (Hempen and Brion, 1996; Silva et al., 2017) and Huntington’s disease (Dompierre et al., 2007), which would be in line with this supposed reduction in stability, more recent work suggests that patients with Alzheimer’s disease may instead have increased levels of modified (e.g., acetylated, detyrosinated, and ∆2) tubulin (Martínez-Hernández et al., 2022; Peris et al., 2022; Vu et al., 2017; Zhang et al., 2015a), which is more likely indicative of an increase in microtubule stability and a lack of dynamicity. This could help to explain why these microtubule stabilizers have failed in clinical trials. Furthermore, tau, which allows dynamic microtubules to have long, labile ends (Qiang et al., 2018) (see also “*Characteristics of dynamic microtubules*”), is sequestered into tangles in tauopathies such as Alzheimer’s disease (Lee et al., 2001); consequently, its lower binding to microtubules may reduce the population of dynamic microtubules. These findings all indicate that a reduction of dynamic microtubules may be an important factor in neurodegenerative diseases and perhaps drugs that aim to increase microtubule dynamicity (rather than stability) should be tested for the treatment of these diseases.

Interestingly, lower levels of MAP6, a key microtubule stabilizer, have been reported in autistic patients (Wei et al., 2016) and the deletion of MAP6 produced cognitive defects in mice (Andrieux et al., 2002; Fournet et al., 2012). This suggests that a decrease in microtubule stability might underlie neurodevelopmental (rather than neurodegenerative) disorders. Similarly, knockdown or inhibition of VASH1/VASH2-SVBP, and to a lesser extent knockout of MATCAP (all enzymes responsible for detyrosination), lead to neurodevelopmental abnormalities and differentiation defects in cultured neurons (Aillaud et al., 2017; Landskron et al., 2022). On the other hand, TTL knockout mice, which die perinatally, also show disorganized neural networks and cultured TTL knockout neurons have defects in polarization and neurite outgrowth (Erck et al., 2005). Thus, an imbalance of tubulin tyrosination and detyrosination in either direction can affect neuronal development. This suggests that restoring the balance of different microtubule subsets in neurons might be one way to counteract these diseases, but which subset should be targeted for treatment is complex and likely disease- and developmental stage-specific.

**FIGURE 6:**
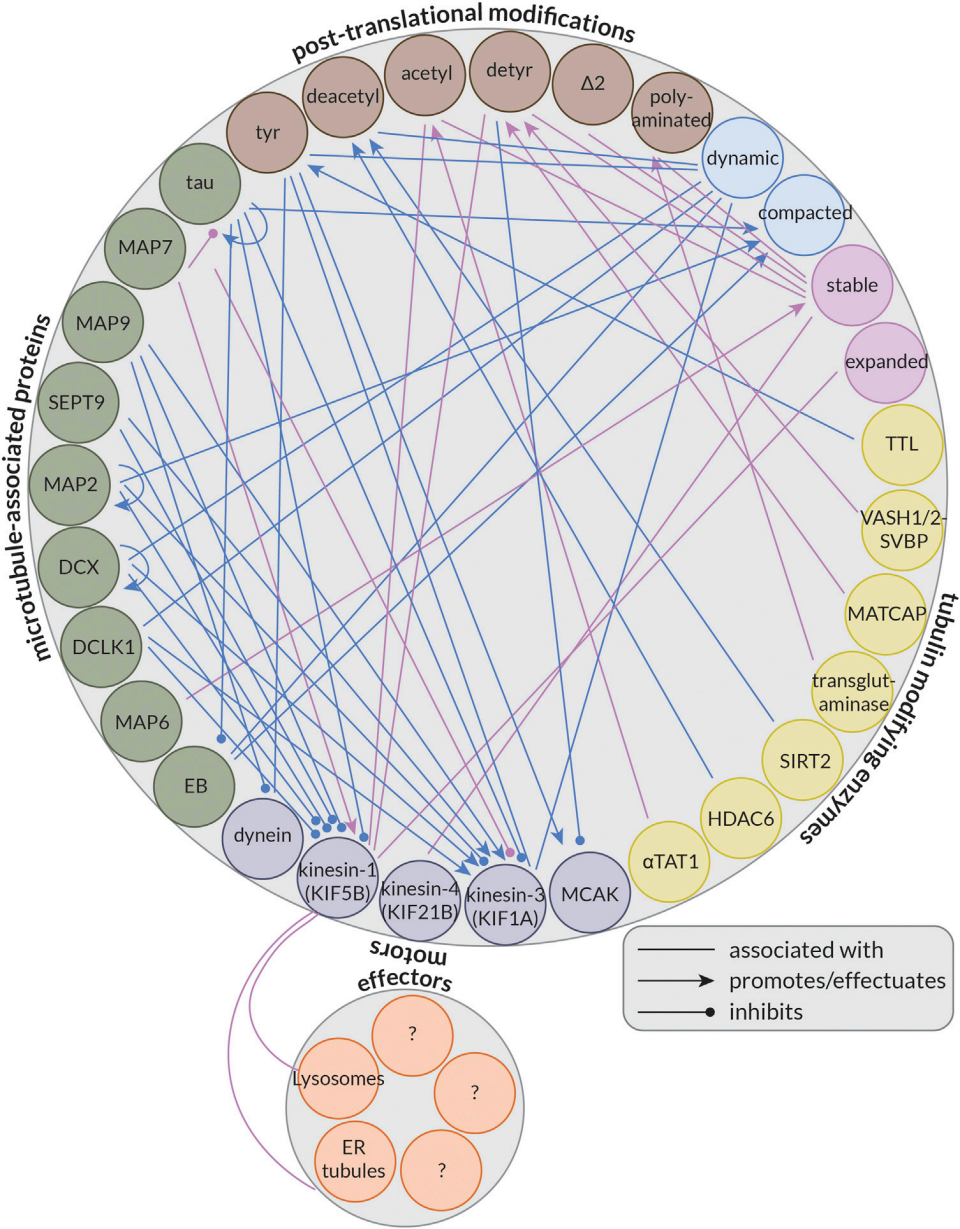
The interplay between different aspects composing microtubule subsets in neurons. A map showing some of the interactions that have been observed for different MAPs, PTMs, tubulin-modifying enzymes, motors, and effectors including which of these factors are associated with, inhibit, or promote others or themselves (cooperativity). Blue lines indicate that the interaction likely takes place on dynamic microtubules. Magenta lines indicate that the interaction likely takes place on stable microtubules. Note that for kinesin-1 there are many inhibitory MAPs that are (likely) associated with dynamic microtubules, preventing it from using these as tracks, whereas for kinesin-3, some of these MAPs are not or less inhibitory, permitting it to use these as tracks. This in turn impacts whether the respective cargoes are associated with stable or dynamic microtubules. See text and Table 1.

In addition to issues determining whether drugs that increase microtubule stability or dynamicity should be used for any given disease, many drugs targeting the microtubule cytoskeleton have issues penetrating the blood-brain barrier or cause unwanted side effects (Varidaki et al., 2018). The latter could be because these drugs do not selectively impact neurons and could thus promote an imbalance in the relative amounts of stable and dynamic microtubules in other cell types. For example, unlike neurons, many cells in our body do continue to divide during our lifetime and the mitotic spindle also requires both stable and dynamic microtubules, as indicated by the distinct PTMs found on different microtubules within the spindle (Barisic et al., 2015). For example, upon treatment with a microtubule stabilizing agent, the balance of microtubule stability in these cells would also be shifted. Moving forward, it would thus be helpful to design or repurpose drugs for new targets involved in regulating microtubule stabilization that are perhaps more specific to neurons. Furthermore, to fully appreciate the potential effects of these drugs, we need to carefully study the interplay between the different aspects discussed in this review—namely, the tubulin code, the MAP code, microtubule orientation, and microtubule dynamicity or stability—and how they collectively exist on various microtubules in the cell, subdividing these into subsets that fulfill unique roles and are preferentially used by different motor proteins. To date, the influence of most of these features have been studied independently from each other, but of course, none of them exist in isolation in the cell (Figure 6; Table 1).

**TABLE 1:** Proposed preferences of MAPs and tubulin-modifying enzymes in neurons.

Protein (modification)	Inferred subset preference	Evidence
Kinesin-1/KIF5A,B,C	Stable (expanded)	Prefers acetylated microtubules in COS-7 cells and rat hippocampal neurons (Cai et al., 2009; Farías et al., 2015; Tas et al., 2017; Jansen et al., 2023); can expand the microtubule lattice (Peet et al., 2018); a kinesin-1 rigor localizes specifically to microtubules with an expanded lattice in U2OS cells (de Jager et al., 2022)
Kinesin-4/KIF21B	Stable	Moves cargo with a retrograde bias in PEX assays in rat hippocampal neurons unless plus-end-out microtubules are stabilized (Masucci et al., 2021)
Dynein	Dynamic	Cofactor p150^Glued^ binds preferentially to tyrosinated microtubules in fibroblasts and *in vitro* (Peris et al., 2006; McKenney et al., 2016)
Kinesin-3/KIF1A	Dynamic	Rigor KIF1A is ∼2-fold enriched on tyrosinated microtubules compared to acetylated microtubules in soma of neurons and COS-7 cells (Tas et al., 2017)
Kinesin-13/MCAK/KIFA	Dynamic	Prefers tyrosinated microtubules in mouse embryonic fibroblasts and *in vitro* (Peris et al., 2009)
MAP7	Stable	Potently recruits and activates kinesin-1 *in vitro* and in HeLa cells (Monroy et al., 2018; Hooikaas et al., 2019; Ferro et al., 2022)
MAP7D1	Stable	MAP7D1 localizes to some sort of subset in HeLa cells; recruits and activates kinesin-1 (Hooikaas et al., 2019)
MAP7D2	Stable	Localizes to the axon initial segment and helps kinesin-1 traffic into the axon of rat hippocampal neurons (Pan et al., 2019)
MAP7D3	Stable	Recruits and activates kinesin-1 *in vitro* and in HeLa cells (Hooikaas et al., 2019)
MAP6	Stable	Stabilizes microtubules against cold and nocodazole-induced depolymerization in mouse (embryonic) fibroblasts, HeLa cells, and neurons (Bosc et al., 2003; Delphin et al., 2012; Cuveillier et al., 2020); resists tubulin compaction leading to coiled microtubules *in vitro* (Cuveillier et al., 2020)
MAP4	Stable?	Does not compact microtubule lattice *in vitro* (Siahaan et al., 2022); can bind microtubule at the same time as kinesin-1 (Shigematsu et al., 2018)
MAP9	Dynamic?	No direct evidence; inhibits kinesin-1, permits kinesin-3 motility *in vitro* (Monroy et al., 2020)
DCX	Dynamic	Involved in nucleating microtubules and stabilizing nucleus *in vitro*, and tracks growing microtubule ends (Bechstedt and Brouhard, 2012); one microtubule-binding pseudorepeat preferentially associates with GTP/GDP-Pi-tubulin, the other with GDP-tubulin (Manka and Moores, 2020)
DCLK1	Dynamic	Preferentially decorates dynamic microtubules (esp. near cell periphery) and is excluded from detyrosinated microtubules in COS-7 cells (Lipka et al., 2016)
MAP2	Dynamic (compacted)	Locally compacts microtubule lattice *in vitro* (and thus likely prefers compacted microtubules) (Siahaan et al., 2022)
Tau	Dynamic (compacted)	Locally compacts microtubule lattice and prefers GDP over GMPCPP microtubules *in vitro* (Tan et al., 2019; Siahaan et al., 2022); allows dynamic microtubules to have long, labile ends *in vitro* and in axons (Qiang et al., 2018); enriched in the axon and helps maintain dynamic microtubule mass there (Qiang et al., 2018)
EB	Dynamic (compacted)	Associated with growing plus-ends of microtubules in all cell types tested; compacts microtubule lattice *in vitro* (LaFrance et al., 2022; Zhang et al., 2018b)
SEPT9	Dynamic?	No direct evidence; inhibits kinesin-1, activates kinesin-3 motility *in vitro* and localizes to the proximal dendrites (Karasmanis et al., 2018)
αTAT1 (acetylation)	Stable	No direct evidence for preference of enzyme; stable microtubules (with long half-life that do not incorporate micro-injected tubulin) are acetylated (Schulze et al., 1987)
HDAC6 (deacetylation)	Dynamic (dimers)	Preferentially (only) acts on soluble tubulin dimers (Prota et al., 2013; Skultetyova et al., 2017), and this deacetylated tubulin can be reincorporated into dynamic microtubules; activity might be impeded in Taxol-treated cells (Xiao et al., 2006)
SIRT2 (deacetylation)	Dynamic?	No direct evidence for preference of enzyme (Schulze et al., 1987); dynamic microtubules are largely deacetylated and stable microtubules are largely acetylated
VASH1/2-SVBP (detyrosination)	Stable	No direct evidence for preference of enzymes; stable microtubules are not tyrosinated; stable microtubules are acetylated and microtubules that are acetylated are usually also detyrosinated (Schulze et al., 1987); VASH1-SVBP and VASH2-SVBP detyrosinate different (regions of) microtubules in permeabilized mouse embryonic fibroblasts (Ramirez-Rios et al., 2022)
MATCAP (detyrosination)	Stable	No direct evidence for preference of enzymes; stable microtubules are not tyrosinated (Schulze et al., 1987)
TTL (tyrosination)	Dynamic (dimers)	Preferentially acts on soluble tubulin dimers (Wehland and Weber, 1987); stable microtubules are not tyrosinated (Schulze et al., 1987); tyrosinated microtubules undergo microtubule dynamics in U2OS and HEK cells (Kesarwani et al., 2020)
Transglutaminase (polyamination)	Stable	No direct evidence for preference of enzyme; cold-stable microtubules in neurons are enriched in poly-aminated tubulin (Song et al., 2013)

A table showing which players we believe are likely found on stable microtubules with expanded lattices and dynamic microtubules with compacted lattices. Indicated are the name of the MAP, motor protein or tubulin modifying enzyme, as well as what evidence suggests that it resides on, preferentially uses or preferentially modifies this subset.

Additionally, it is unclear if microtubules have a fairly uniform identity along their length, as there has been evidence both in support of this idea (Schulze and Kirschner, 1987; Katrukha et al., 2021) and suggesting otherwise (Baas and Black, 1990; Baas and Ahmad, 1992; Ahmad et al., 1993; Qiang et al., 2018). To answer questions such as this and decipher the precise architecture of the neuronal microtubule cytoskeleton, more high-resolution imaging studies are required. Specifically, there is a need for studies simultaneously investigating multiple properties of single microtubules (e.g., if a given microtubule is acetylated and detyrosinated or acetylated and MAP6-positive). These experiments are only starting to become possible with advances in microscopy that facilitate the mapping of single microtubules in neurons including ExM (Chen et al., 2015; Damstra et al., 2022; Gambarotto et al., 2019; Tillberg et al., 2016), MINFLUX (Gwosch et al., 2020; Schmidt et al., 2021), and multi-colour STORM (Andronov et al., 2021; Lampe et al., 2012; Siemons et al., 2022; Winterflood et al., 2015; Zhang et al., 2020).

These types of studies simultaneously investigating two or more properties of microtubules on a per-microtubule basis will also require us to image a wider variety of MAPs, PTMs, tubulin isotypes, and motors to get a more comprehensive picture of the microtubule network architecture and how it is used in neurons. For example, while multiple studies have focused on high resolution imaging of acetylated and (de)tyrosinated tubulin, as well as the effects of tau and MAP7 on the motility of kinesin-1, kinesin-3, and dynein, much less is known about the effects of other prominent neuronal MAPs (e.g., MAP1a, MAP1b, MAP2, MAP4, MAP6, and MAP9) or about how other tubulin PTMs (e.g., polyamination, polyglutamylation, polyglycylation, Δ2, and methylation) are organized within neurites, as well as how different PTMs and MAPs influence members of the kinesin family beyond KIF1A (kinesin-3) and KIF5B (kinesin-1). Furthermore, high-resolution mapping of the localization of tubulin isotypes and whether these partially segregate to different microtubules will hopefully become possible with the help of techniques such as ORANGE (Willems et al., 2020). For example, these isotypes may differ in their dynamicity or stability (Chew and Cross, 2023). Further advances in labeling technologies and the development of live-cell markers for different microtubule properties, as well as developments in light and electron microscopy will surely advance our continued study and comprehensive mapping of microtubule subsets including their MAPs, PTMs, isotypes, orientations, stability, and motor proteins, allowing us to fill in the blanks in our atlas of the neuronal microtubule cytoskeleton.

## Glossary

αK40: α-tubulin lysine 40
αTAT1: α-Tubulin AcetylTransferase 1
βQ15: β-tubulin glutamine 15
CCP1: Cytosolic CarboxyPeptidase 1
CLIP170: Cytoplasmic Linker Protein 170
DCLK1: DoubleCortin-Like Kinase 1
DCX: DoubleCortin
EB: End-Binding
EM: Electron Microscopy
ExM: Expansion Microscopy
FKBP12: 12-kDa FK506 Binding Brotein
FRB: FKBP-Rapamycin Binding
γTuRC: γ-Tubulin Ring Complex
GDP: Guanosine 5′-DiPhosphate
GMPCPP: Guanosine-5′-[(α,β)-methyleno]triphosphate
GTP: Guanosine-5′-TriPhosphate
HAUS: Human AUgmin complex Subunit
HDAC6: Histone DeACetylase 6
MAP: Microtubule-Associated Protein
MATCAP: Microtubule-Associated Tyrosine CArboxyPeptidase
MCAK: Mitotic Centromere-Associated Kinesin
MINFLUX: MINimal photon FLUXes
MTOC: MicroTubule Organizing Center
PAINT: Point Accumulation for Imaging in Nanoscale Topography
PRC1: Protein Regulator of Cytokinesis 1
PTM: Post-Translational Modification
RPE cells: Retinal Pigment Epithelial cells
RTN4: ReTiculoN 4
SEPT9: SEPTin 9
SIRT2: SIRTuin 2
StableMARK: Stable Microtubule-Associated Rigor Kinesin
STED: STimulated Emission Depletion
STORM: STochastic Optical Reconstruction Microscopy
SVBP: Small Vasohibin Binding Protein
+TIP: Plus-end tracking protein
TIRF: Total Internal Reflection Fluorescence
TREx: Tenfold-Robust Expansion
TTL: Tubulin Tyrosine Ligase
TTLL: Tubulin Tyrosine Ligase Like
VASH: VASoHibin
