# Corrigendum: Cellular cartography: towards an atlas of the neuronal microtubule cytoskeleton

**DOI:** 10.3389/fcell.2023.1232120

**Published:** 2023-06-15

**Authors:** Malina K. Iwanski, Lukas C. Kapitein

**Affiliations:** Cell Biology, Neurobiology and Biophysics, Department of Biology, Faculty of Science, Utrecht University, Utrecht, Netherlands

**Keywords:** microtubules, neurons, microtubule stability, post-translational modifications, microtubule-associated proteins, microscopy

In the published article, there was an error in (Figure 5) as published. The text in the middle of the figure read “stable microtubules ∼2/3 plus-end out”, while this should read “stable microtubules ∼2/3 minus-end out”. The corrected (Figure 5) and its caption appear below.

**FIGURE 5:**
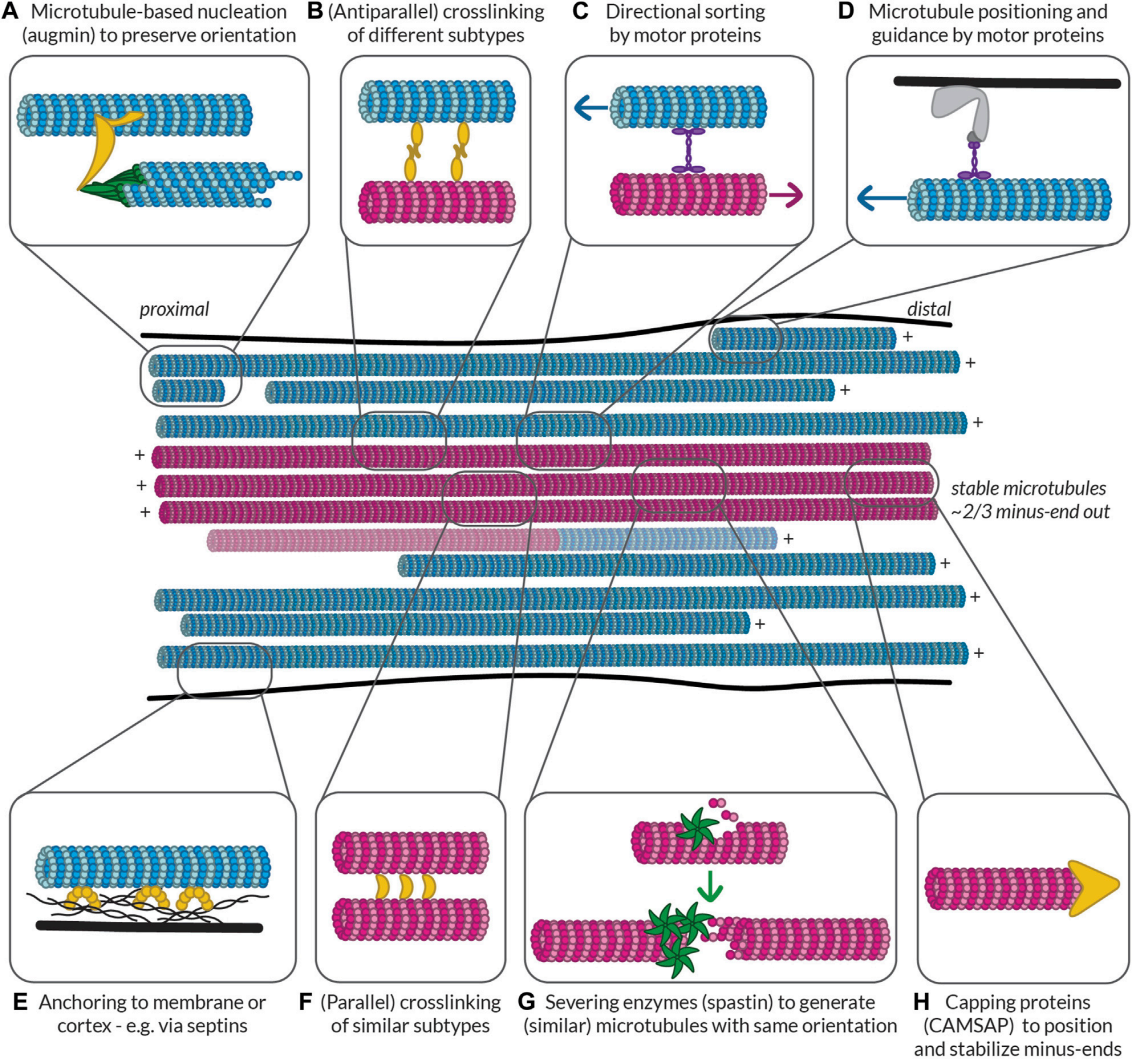
Possible ways to build and maintain (partially) segregated dendritic microtubule subsets. Some of the mechanisms that could be at play to help establish and maintain subset identity in terms of orientation, PTMs, and MAP decoration. Controlled nucleation, such as with **(A)** augmin and **(G)** off of microtubule seeds generated by spastin-mediated severing can help preserve local microtubule orientation. Different mechanisms can then help **(F)** bundle similar microtubules via (parallel) crosslinkers, **(B)** link different bundles via (antiparallel) crosslinkers, and **(C)** keep uniform orientation within bundles by pushing out microtubules of the opposite orientation. Microtubules can also be properly positioned based on their orientation, PTMs, or MAPs via **(D)** motor proteins anchored to the membrane or cortex or **(E)** other MAPs such as septins anchored to the membrane or cortex. Finally, **(H)** capping proteins such as CAMSAPs can help to position the (minus-)ends of microtubules properly relative to, e.g., branch points or synapses. All these proteins could recognize the relative orientation of microtubules or the complement of MAPs and PTMs that they bear. Furthermore, by bundling similar microtubules, tubulin-modifying enzymes and MAPs could move between microtubules to help maintain subset identity. Magenta microtubules are stable (i.e., have an expanded lattice and are decorated with the associated MAPs such as MAP6 and PTMs such as acetylation and detyrosination), while blue microtubules are dynamic (i.e., have a compacted lattice and are decorated with MAPs such as EB and are tyrosinated). There is a possibility that some microtubules are mixed and have a stable base and a dynamic end, so such a microtubule is also shown.

The authors apologize for this error and state that this does not change the scientific conclusions of the article in any way. The original article has been updated.

